# Quasihexagonal Platinum Nanodendrites Decorated over CoS_2_‐N‐Doped Reduced Graphene Oxide for Electro‐Oxidation of C1‐, C2‐, and C3‐Type Alcohols

**DOI:** 10.1002/advs.202105344

**Published:** 2022-01-20

**Authors:** Natarajan Logeshwaran, Iyyappa Rajan Panneerselvam, Shanmugam Ramakrishnan, Ramasamy Santhosh Kumar, Ae Rhan Kim, Yan Wang, Dong Jin Yoo

**Affiliations:** ^1^ Graduate School Department of Energy Storage/Conversion Engineering (BK21 FOUR) Hydrogen and Fuel Cell Research Center Jeonbuk National University Jeonju Jeollabuk‐do 54896 Republic of Korea; ^2^ Department of Mechanical Engineering University of Nevada, Reno Reno NV 89557 USA; ^3^ Department of Life Science Jeonbuk National University Jeonju Jeollabuk‐do 54896 Republic of Korea

**Keywords:** density functional theory, ethylene glycol oxidation, glycerol oxidation, methanol oxidation, Pt‐nanodendrites

## Abstract

The development of efficient and highly durable materials for renewable energy conversion devices is crucial to the future of clean energy demand. Herein, cage‐like quasihexagonal structured platinum nanodendrites decorated over the transition metal chalcogenide core (CoS_2_)‐N‐doped graphene oxide (PtNDs@CoS_2_‐NrGO) through optimized shape engineering and structural control technology are fabricated. The prepared electrocatalyst of PtNDs@CoS_2_‐NrGO is effectively used as anodic catalyst for alcohol oxidation in direct liquid alcohol fuel cells. Notably, the prepared PtNDs@CoS_2_‐NrGO exhibits superior electrocatalytic performance toward alcohol oxidation with higher oxidation peak current densities of 491.31, 440.25, and 438.12 mA mg_pt_^–1^ for (methanol) C1, (ethylene glycol) C2, and (glycerol) C3 fuel electrolytes, respectively, as compared to state‐of‐the‐art Pt‐C in acidic medium. The electro‐oxidation durability of PtNDs@CoS_2_‐NrGO is investigated through cyclic voltammetry and chronoamperometry tests, which demonstrate excellent stability of the electrocatalyst toward various alcohols. Furthermore, the surface and adsorption energies of PtNDs and CoS_2_ are calculated using density functional theory along with the detailed bonding analysis. Overall, the obtained results emphasize the advances in effective precious material utilization and fabricating techniques of active electrocatalysts for direct alcohol oxidation fuel cell applications.

## Graphical abstract

This work demonstrates synthesis of quasihexagonal platinum nanodendrites on transition metal chalcogenide core (CoS_2_)‐N‐doped graphene oxide (PtNDs@CoS_2_‐NrGO) through optimized shape engineering and structural control technology. The PtNDs@CoS_2_‐NrGO electrocatalyst shows superior electrocatalytic performance toward alcohol oxidation with higher oxidation peak current densities of 491.31, 440.25, and 438.12 mA mg_pt_^–1^ for (methanol) C1, (ethylene glycol) C2, and (glycerol) C3 fuel electrolytes, respectively.

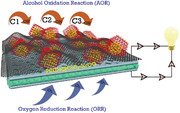

## 1 Introduction

The research on energy storage and conversion has widely focused on sustainable energy technologies such as water electrolysis, fuel cells, solar power panels, metal–air/ion batteries, and wind power.^[^ ^1^, ^2^, ^3^, ^4^, ^5^ ^]^ Among diverse renewable energy devices, fuel cells, especially liquid alcohol fuel cells (AFCs), have received much attention due to their high energy density, minimal environmental hazards, compact design, quick start‐up time, low operating temperature, and ease of handling.^[^ ^6^, ^7^ ^]^ Significant research has been focused primarily on the commercialization of direct methanol (C1) fuel cells (DMFCs) due to their high energy density, rich supply, renewability, and easy affordability. In the same way, C2‐type ethylene glycol (EG) (C_2_H_6_O_2_)^[^ ^8^, ^9^, ^10^ ^]^ and C3‐type glycerol (C_3_H_8_O_3_)^[^ ^11^, ^12^, ^13^, ^14^ ^]^ alcohols, which have high theoretical energy density and lower volatility, are also alternatives for the commercialization of multiple types of AFCs. As a pivotal component, the anode electrode plays a key role in liquid fuel oxidation in AFCs. The rational design of the anode electrode and its fabrication mainly depends on low‐cost material selection, carbon monoxide tolerance capability, multiple fuel flexibility (C1, C2, and C3 types of fuel), and prolonged stability. Additionally, low oxidation dynamics, fuel cross‐over through electrolytes, and polarizing carbonaceous intermediates such as —COO, —CO, and —COOH form on catalyst surfaces, while C—C bond breakage occurs during the oxidation reaction. All of these factors, considered together, have significantly hampered the commercialization of AFCs.^[^ ^15^ ^]^

Platinum‐ruthenium/carbon (Pt‐Ru/C) based alloys are considered state‐of‐the‐art anode electrocatalysts in AFCs due to their faster reaction kinetics, enlarged active surface area, and rational nanosized design. Associated problems of high price, easy poisoning of CO intermediate, and unsatisfactory durability have prompted researchers to develop an efficient and durable Pt‐based anode electrocatalyst in AFCs. Transition metal dichalcogenides (TMDs) have recently emerged as attractive catalytic materials to couple with Pt due to their cost‐effective, sufficient element reserves, and enlarged active surface areas. Interestingly, cobalt pyrites (CoS_2_, CoSe_2_) have been widely used in renewable energy‐oriented devices due to their low Gibbs adsorption energy, numerous electroactive sites, and sufficient distinctive d‐orbital electronic structures. TMD metal sulfide‐CoS_2_ generates active OH^−^ from water molecules, which can prevent the formation of poisonous CO layers on the Pt surface during the continuous alcohol oxidation process.^[^ ^16^, ^17^, ^18^ ^]^ TMDs also require heteroatom (nitrogen (N), sulfur (S), phosphorus (P), and fluorine (F)) doped highly conductive carbon substrates such as carbon black, graphene nanotube/fiber, and reduced graphene oxide (GO) to facilitate faster electron transport during alcohol oxidation reaction.^[^ ^19^, ^20^ ^]^ In particular, heteroatom‐doped, reduced graphene oxides can stimulate faster reaction kinetics and form strong interfacial interactions with Pt and CoS_2_.

Engineering shape and size‐controlled nanoscale material preparations are of significant interest for commercialization of electrocatalysts. Numerous attempts to structurally tune Pt electrocatalyst have been developed, such as generating core‐shell, nanowires, nanodendrites and nanosphere structures. Among these, nanodendrites orientation of Pt particle (PtND) preparation preserves higher catalytic active surface area, which offers electron‐rich corners of structures.^[^ ^21^, ^22^ ^]^ Besides, fabricating PtNDs (platinum nanodendrites) on the TMD with an optimized structural morphology is a challenging task. Using ecofriendly structural directing reductant agents is highly recommended for the synthesis of Pt nanodendrites on catalyst surfaces. Pluronic (PEO_100_‐PPO_65_‐PEO_100_) F‐127 block copolymer can be used as both a structure‐directing agent and a surfactant in wet chemical synthesis methods.^[^ ^23^ ^]^ Frank‐van der Merwe growth (FM growth) model is one of the effective crystal formation techniques. In which the crystal lattice are well arranged with layer‐by‐layer mode.^[^ ^24^ ^]^ According with FM growth model this negatively charged F‐127 contains hydrophobic polypropylene oxide (PPO) and polyethylene oxide (PEO), in which PPO might extensively adsorb on TMD surfaces, while PEO can act as a reductant for the positive charged Pt ions, allowing them to grow in a layer‐by‐layer mode to form Pt nanodendrites. In addition, using one softer, reducing agent (ascorbic acid) can promote the atomic growth of nanodendrites. Also, this can limit the aggregation growth of Pt nanodendrites arms over TMD surfaces. These dual reducing methods create novel 3D cage‐like platinum nanodendrites on TMD surfaces. This can improve the electrocatalytic activity and structural retention ability of electrocatalysts after long‐term cyclic stability evaluations.

Inspired by the Frank‐van der Merwe growth model of electrocatalyst preparations, we have designed cage‐like quasi hexagonal 3D Pt nanodendrites (PtNDs) on cobalt sulfides (CoS_2_) by a uniform shape‐engineering technique. Using an agile one‐pot hydrothermal synthesis followed by a wet‐chemical reflux method, PtNDs@CoS_2_ were prepared on nitrogen‐doped reduced graphene oxide where Pt were nanodendrites formed as assisted by Pluronic F‐127 block copolymer and ascorbic acid. The Pt nanodendrites arms, with an average size of 3–4 nm were homogeneously grown on the CoS_2_‐NrGO surfaces. The optimized PtNDs@CoS_2_‐NrGO electrocatalyst exhibited excellent electrocatalytic performance toward methanol, ethylene glycol, and glycerol with higher mass activities of 491.31, 440.25, and 438.12 mA mg_pt_^–1^, and smaller Tafel slopes of 55.52, 56.73, and 61.93 mV dec^–1^, respectively, in a 0.5 m H_2_SO_4_ medium. The prepared Pt‐NrGO and commercial Pt‐C were also tested for comparison. In addition, we performed density functional theory (DFT) calculations on Pt (111) and CoS_2_ (200) surfaces and the results reveal that the CoS_2_ (200) surface is more thermodynamically favorable than the Pt (111) surface. Therefore, the surface of CoS_2_ (200) is readily available for alcohol adsorption than Pt (111) surfaces for catalytic reactions. To gain a better understanding of the adsorption/desorption behavior of methanol on the CoS_2_ (200) surface, we calculated the projected crystal orbital Hamilton population (pCOHP) and integrated COHP (ICOHP) using the local‐orbital basis suite toward electronic‐structure reconstruction (LOBSTER) code. These novel rational quasihexagonal 3D‐structured Pt nanodendrites on transition metal dichalcogenides electrocatalysts are expected to be commercialized in numerous future electrochemical applications.

## 2 Results and Discussion

### 2.1 Structural and Morphological Studies

**Scheme 1:**
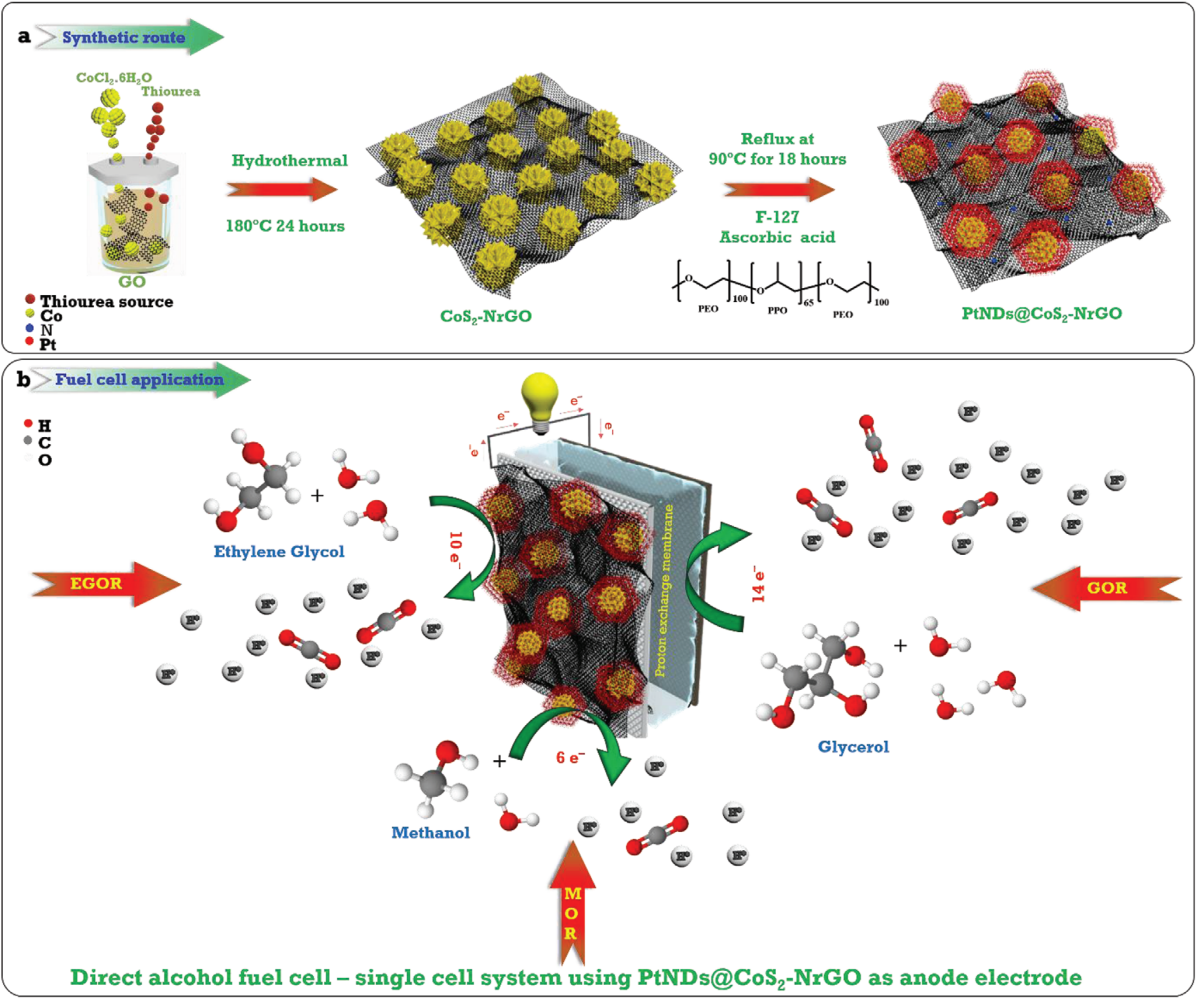
a) Schematic illustration for the synthesis process of CoS_2_ on nitrogen‐doped reduced graphene oxide sheets and the subsequent Pt nanoparticles’ reduction on the CoS_2_ nanoparticles by the chemical wet reflex process. b) Proposed mechanism of methanol, ethylene glycol, and glycerol alcohol oxidation reaction on typically prepared PtNDs@CoS_2_‐NrGO electrocatalysts.

**Figure 1:**
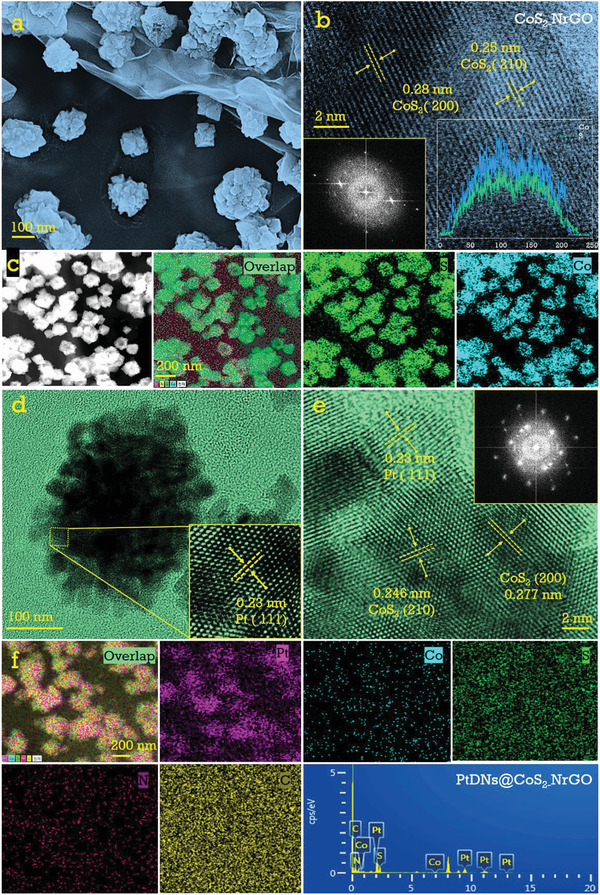
Representative physical morphology of a) high‐magnification FE‐SEM image of CoS_2_‐NrGO, b) high‐magnification HR‐TEM image of CoS_2_‐NrGO (insets represents corresponding fast Fourier transform patterns and line scan spectra of Co, S), c) STEM electron image of the CoS_2_‐NrGO electrocatalysts and their corresponding HAADF‐STEM color mapping of the Co and S elements, d,e) lower and higher magnification HR‐TEM images of PtNDs@CoS_2_‐NrGO electrocatalysts (insets represent lattice fringes of Pt (111) plane) and (insets of (e) represent FFT patterns of PtNDs@CoS_2_), f) HAADF‐STEM elemental color mapping of the Pt, Co, S, N, C elements and their corresponding dispersion spectra.

**Scheme** **1**a illustrates an agile, continuous two‐step hydrothermal reduction, and reflux synthetic methodology of highly dispersed low Pt nanodendritic growth on CoS_2_‐nitrogen‐doped graphene oxide substrates. In the first step, TMD CoS_2_ nanoparticles encased in highly conducting active nitrogen‐doped graphene oxide were synthesized using the one‐pot hydrothermal method. Then, a subsequent reduction process by a wet chemical reflection method was followed to attain a homogeneous distribution of low weight percentage Pt nanodendrites. Ascorbic acid and Pluronic F‐127 block copolymer (PEO_100_PPO_65_PEO_100_) were used as reducing and structure‐directing agents, respectively. Scheme 1b illustrates the proposed mechanism of alcohol oxidation by a typically prepared PtNDs@CoS_2_‐NrGO electrocatalyst where the direct oxidation reaction pathways over the three C‐type fuel molecules are described. The morphology analysis of the prepared electrocatalyst was carried out using field emission microscopy (FE‐SEM) and high‐resolution transmission electron microscopy (HR‐TEM). **Figure** **1**a shows an FE‐SEM image of typically prepared CoS_2_‐NrGO, in which the hydrothermal process reduces GO to rGO on adding thiourea. Thiourea can act as both a sulfur (S) and nitrogen (N) source that gets decomposed and reduces the rGO, thereby making bonds with Co^2+^ ions during the high‐temperature hydrothermal synthesis. Subsequently, S^2‐^ ions from the thiourea were slowly reduced on Co^2+^/nitrogen‐doped rGO. Where CoS_2_ particles with an average diameter of ≈200 nm are uniformly encased in 2D‐shaped reduced graphene oxide layers. As a result, the consistent growth of cobalt nanoparticles between graphene layers has been confirmed. Moreover, this kind of architecture promotes faster reaction kinetics and high conductivity of the electrocatalyst. Figure 1b shows an HR‐TEM image of CoS_2_‐NrGO that reveals the formation of high crystalline cobalt sulfides with lattice fringes (210) and (200) and interplanar spacings of 0.25 and 0.28 nm, respectively (insets show the fast Fourier transform (FFT) pattern of the respective image, which reveals the hexagonal crystalline nature of the CoS_2_‐NrGO electrocatalyst).

**Figure 2:**
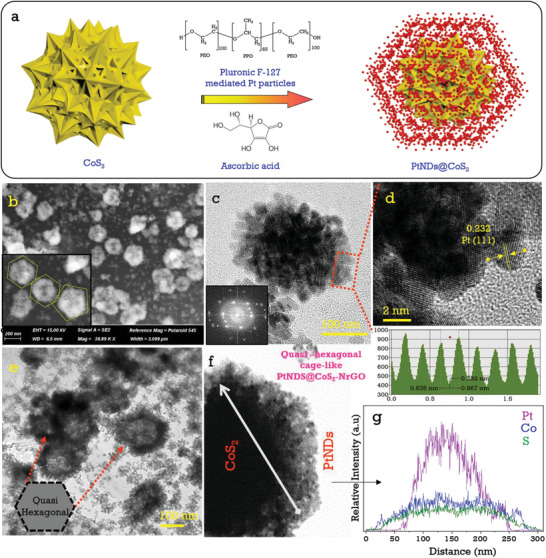
a) Proposed schematic illustration of Pt nanodendrites (PtNDs) material preparation on CoS_2_ associated with Pluronic F‐127 (PEO_100_‐PPO_65_‐PEO_100_) block copolymer and ascorbic acid (yellow ball represents‐CoS_2_, red dots represents‐PtNDs), b) FE‐SEM images of PtNDs@CoS_2_‐NrGO, c,d) high‐resolution TEM images evidence of PtNDs formation on CoS_2_ nanoparticles, e) representative CS‐TEM image of quasi hexagonal shape like PtNDs@CoS_2_‐NrGO, f,g) representative HAADF‐STEM images and line scanning spectra of Pt, Co, and S element in PtNDs@CoS_2_‐NrGO electrocatalysts.

Figure 1c depicts high‐resolution HAADF‐STEM color mapping images of the CoS_2_‐NrGO electrocatalyst, revealing that Co and S elements are developed homogeneously on the NrGO. These findings confirm the formation of CoS_2_ particles on the NrGO substrate. Pt nanodendrites (PtNDs) were then uniformly coated on CoS_2_‐NrGO surfaces using the wet chemical reflux method. This was accomplished by reducing the Pt precursor (K_2_PtCl_4_) with ascorbic acid (C_6_H_8_O_6_) and F‐127 (PEO_100_‐PPO_65_‐PEO_100_) block copolymer at 90 ^◦^C for 18 h, which corresponded to the following reaction: K_2_PtCl_4_ + C_6_H_8_O_6_ → Pt^0^ + C_6_H_6_O_6_ + 2KCl + 2HCl.^[^ ^21^ ^]^ The aforementioned reaction promotes the formation of Pt nanodendrites over the CoS_2_ surfaces in the form of quasihexagonal cage‐like 3D structures. As shown in Figure 1d, the HR‐TEM image of hexagonal cage‐like PtNDs in which the insets demonstrate that the Pt (111) particles wrapped the CoS_2_ surfaces where no more agglomerations were found, and Figure 1e depicts a highly crystalline HR‐TEM image of crystal lattice fringes with d‐spacing values of 0.246 nm and 0.277 nm, respectively, corresponding to CoS_2_ (210) and (200) lattice planes. Furthermore, the edges were wrapped with Pt (111) particles, the SAED patterns for which can be found in supporting information (Figure S1, Supporting Information). The high‐resolution HAADF‐STEM color mapping and elemental energy dispersive X‐ray (EDX) images in Figure 1f indicate a uniform distribution of Pt nanoparticles over the CoS_2_‐NrGO surfaces and justify the successful formation of the PtNDs@CoS_2_‐NrGO electrocatalyst. A schematic illustration of the 3D Pt nanodendrites formation over the CoS_2_ surface is shown in **Figure** **2**a. Pluronic amphiphilic block copolymer contains hygrophoroid PPO (polypropylene oxide), which can adsorb at the Pt metal particles, creating PPO micelles to prevent agglomeration of Pt nanodendrites.^[^ ^25^, ^26^, ^27^ ^]^ Polyethylene oxide (PEO) can act as a reducing agent for positively charged Pt ions and it grow in a layer‐by‐layer mode (i.e., the Frank‐van der Merwe growth mode) to form Pt nanodendrites over the CoS_2_ surface. FE‐SEM image in Figure 2b shows the quasihexagonal shapes of PtNDs@CoS_2_‐NrGO, in which some few Pt particles were deposited on the graphene surfaces. This architecture can improve the extended catalytic active surface area and also dendritic structures have a high number of catalytically active atoms at the edge and corner, allowing for plenty of catalytic sites and enough absorption sites. HR‐TEM images in Figure 2c–d show that formation of quasihexagonal cage Pt nanodendrites on CoS_2_‐NrGO and also Pt nanoparticles are coated on the NrGO sheet. The inset image in Figure 2d shows that Pt (111) plane nanodendrites are uniformly formed over the CoS_2_ surfaces. Additional HADDF‐STEM line‐scan mapping evaluation of 3D quasihexagonal cage Pt nanodendrites in Figure 2f,g also supports the formation of PtNDs@CoS_2_‐NrGO. The thermal stability of PtNDs@CoS_2_‐NrGO, CoS_2_‐NrGO, Pt‐NrGO, and commercial Pt‐C is determined by the thermogravimetric curve as shown in Figure S2 (Supporting Information). These compounds exhibit 31.32%, 27.01%, 4.93%, and 21.68% material retention capacity, respectively, in an air environment and reveal that PtNDs cages protect the material during high temperature conditions.

The diffractogram of the synthesized materials were examined using XRD analysis. Figure S3 (Supporting Information) shows the collected XRD patterns of PtNDs@CoS_2_‐NrGO, Pt‐C, Pt‐NrGO, CoS_2_‐NrGO, and NrGO electrocatalysts, respectively. In all the diffraction patterns, peaks around (24–26^◦^) (2*θ*) with a (002) plane account for the presence of graphitic oxide carbon, which is reduced to graphene formed using a step‐by‐step reduction process. Sharp peaks appeared at 32.53°, 36.41°, 40.01°, 46.58°, 55.20°, 60.2°, and 62.82° corresponding to (200), (210), (211), (220), (311), (230), and (321) facets of CoS_2_‐NrGO, in agreement with standard cubic CoS_2_ (JCPDS no. 00‐041‐1471).^[^ ^28^, ^29^ ^]^ After growth of PtNDs on CoS_2_‐NrGO, XRD pattern displays the sharp peaks at 39.83°, 46.35°, 67.64°, 81.37°, and 86.03° correspond to (111), (200), (220), (311), and (222) facets of PtNDs@CoS_2_‐NrGO with respect to the standard cubic Pt (JCPDS no. 65–2868).^[^ ^30^ ^]^ Figure S3 (Supporting Information) shows high‐intensity peaks of PtNDs in PtNDs@CoS_2_‐NrGO, whereas CoS_2_ and NrGO peak intensities were suppressed due to the high crystalline nature of PtND grown on the CoS_2_‐NrGO electrocatalyst.^[^ ^31^ ^]^ This confirms the successful formation of Pt nanodendrites on the CoS_2_‐NrGO surfaces.^[^ ^32^ ^]^

Raman spectrum analysis is an efficient tool to calculate the impact of carbon defects on the electrocatalysts prepared in Figure S4 (Supporting Information). Intensities of the D and G bands were observed around 1345 and 1590 cm^–1^. Furthermore, the *I*_D_/*I*_G_ ratios of GO, NrGO, CoS_2_‐NrGO, Pt‐NrGO, PtNDs@CoS_2_‐NrGO, and Pt‐C electrocatalysts were calculated to be 0.83, 0.99, 1.02, 1.00, 1.04, and 0.99, respectively.^[^ ^33^, ^34^, ^35^ ^]^ The *I*_D_/*I*_G_ ratio of PtNDs@CoS_2_‐NrGO electrocatalysts was higher than those of the other electrocatalysts. This result suggests that the PtNDs formed on the CoS_2_‐NrGO using the wet reflux method are of high quality. Moreover, the surface morphologies were examined using atomic force microscopy (AFM). As shown in Figure S5 (Supporting Information), the topographical height profiles of PtNDs@CoS_2_‐NrGO electrocatalysts were measured in 2D and 3D angle views, exposing the high roughness properties of 3D PtNDs@CoS_2_ on the conducting graphene oxide layers. A few graphene oxide layers exhibited a smooth surface devoid of metal nanoparticles. The roughness (Rf) of the PtNDs@CoS_2_ incorporated into the graphene oxide layers was calculated to be 9.5–13.25. Additionally, we conducted ICP‐OES analyzes to examine the metal percentage evaluations in the as‐prepared electrocatalyst, and this revealed that Pt and Co elements were present at 16.9% and 5.7%, respectively. These results summarize the HAADF‐STEM elemental composition scanning results in Table S3 (Supporting Information).

**Figure 3:**
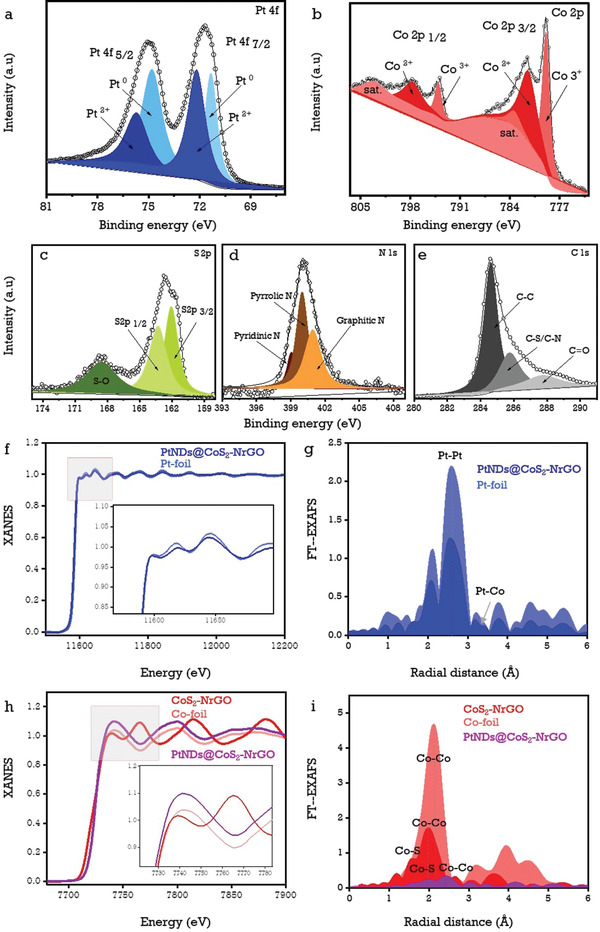
High‐resolution XPS deconvolution spectra of PtNDs@CoS_2_‐NrGO electrocatalyst elements: a) Pt 4f, b) Co 2p, c) S 2p, d) N 1s, and e) C 1s; Pt‐L_3_ edge XANES spectra (f) and FT‐EXAFS spectra (g) of PtNDs@CoS_2_‐NrGO electrocatalyst and Pt‐foil, Co‐K edge XANES spectra (h) and FT‐EXAFS spectra (i) of CoS_2_‐NrGO, PtNDs@CoS_2_‐NrGO and Co‐foil.

The elemental valence states and surface compositions of PtNDs@CoS_2_‐NrGO and CoS_2_‐NrGO electrocatalysts were evaluated using X‐ray photoelectron spectroscopy (XPS). Figure S6 (Supporting Information) depicts the XPS survey spectrum of PtNDs@CoS_2_‐NrGO and CoS_2_‐NrGO electrocatalysts to identify the presence of Pt, Co, S, C, and N elements. Tables S4 and S5 (Supporting Information) show the corresponding elemental information of CoS_2_‐NrGO and PtNDs@CoS_2_‐NrGO electrocatalysts. Further, the Pt 4f high‐resolution XPS spectrum in **Figure** **3**a was deconvoluted into two major doublets of Pt 4f_5/2_, and Pt 4f_7/2_ with binding energy values centered at 74.8, 75.7, 71.3 and 72.1 eV which were attributed to the Pt^0^ and Pt^2+^ states.^[^ ^36^ ^]^ The high‐resolution XPS deconvolution spectra of Co 2p in Figure 3b shows that the Co 2p_3/2_ binding energies for Co^3+^ and Co^2+^ states were 778.9 and 781.5 eV, while the Co 2p_1/2_ binding energies for Co^3+^ and Co^2+^ states were 794.1 and 797.8 eV, respectively.^[^ ^37^ ^]^ The XPS spectra of PtNDs@CoS_2_‐NrGO show that the Co^3+^ (778.9 eV) peak in Co 2p_3/2_ has a slightly higher binding energy (eV) than CoS_2_‐NrGO (Co^3+^ (Co 2p_3/2_): 778.7 eV) as shown in Figure 3b and Figure S7a (Supporting Information). Furthermore, deconvoluted peaks of Co 2p_1/2_ (Co^3+^: 794.1 eV and Co^2+^: 797.8 eV) have lower bonding energy shifted as compared to deconvoluted peaks of Co 2p_1/2_ in CoS_2_‐NrGO (Co^3+^: 795.2 eV and Co^2+^: 798.6 eV). Additionally, we have noticed that there are changes in the peak intensities and shapes of Co 2p_3/2_, Co 2p_1/2_, and satellite peaks after the growth of PtNDs on CoS_2_‐NrGO. The shifting of peaks occurs when the Pt particles interconnect with CoS_2_‐NrGO surfaces, resulting in surface electronic structure changes and delocalization of d‐band center values.^[^ ^16^ ^]^ Meanwhile, the Co^3+^/Co^2+^ ratio for PtNDs@CoS_2_‐NrGO was 0.85, whereas CoS_2_‐NrGO had a ratio of 0.61. This can enhance the overall oxidation capacity of the PtNDs@CoS_2_‐NrGO electrocatalyst,^[^ ^38^ ^]^ as well as promote excellent oxidation capability. The S 2p spectra were deconvoluted further to yield two major peaks at 162.0 and 163.2 eV for S 2p_3/2_ and S 2p_1/2_, respectively^[^ ^39^ ^]^ (Figure 3c). The area under the sulfur oxidation peak surface in PtNDs@CoS_2_‐NrGO was 2.5 times lower than in CoS_2_‐NrGO due to the effective wet‐reflux reduction reaction. These findings are attributed to the formation of metallic platinum nanodendrites on cobalt sulfide surfaces. Furthermore, in Figure 3d, the high‐resolution N1s XPS spectra were deconvoluted into three prominent nitrogen functional group peaks on the graphene surface: pyridinic‐N, pyrrolic‐N, and graphitic‐N at 398.2, 399.2, and 400.1 eV, respectively.^[^ ^39^ ^]^ This finding supports nitrogen doping over the graphene surfaces. The core level C1s XPS spectra were deconvoluted into three major peaks at 284.6, 285.7 and 287.7 eV, which were assigned to the C—C, C—S/C—N, and C═O groups, respectively.^[^ ^40^ ^]^ These values reveal the presence of S and N functional groups on the graphene surface depicted in Figure 3e. The details of XPS deconvolution for the CoS_2_‐NrGO electrocatalyst can be found in the Supporting Information (Figure S7).

To provide an in‐depth understanding of the electronic configuration and surface coordination states of platinum and cobalt elements, X‐ray absorption near‐edge spectroscopy (XANES) and Fourier‐transformed extended X‐ray absorption fine structure (EXAFS) were conducted with references to Pt and Co foils. The near‐edge energy position of PtNDs‐CoS_2_ found with PtNDs@CoS_2_‐NrGO and Pt foil reference is shown in Figure 3f, indicating that Pt shells were dispersed on CoS_2_ cores with a mixture of Pt^0^ and Pt^2+^ states.^[^ ^16^ ^]^ The white line (WL) intensity feature of the Pt‐L_3_ edge and Co‐K edge in the PtNDs@CoS_2_‐NrGO composite and Pt‐foil denoted the increased WL intensity of Pt in PtNDs@CoS_2_‐NrGO, resulting in a slight increase in the oxidation state of Pt. Meanwhile, the intensity of Co‐WL was comparatively higher than that of Pt‐foil due to the better intermetallic electronic configuration of Pt‐Co in PtNDs@CoS_2_‐NrGO,^[^ ^41^ ^]^ which can easily improve the oxidation capacity for alcohol molecules. Figure 3g shows the Fourier‐transformed (EXAFS) spectra of PtNDs@CoS_2_‐NrGO electrocatalysts, revealing Pt‐Co and Pt‐S intercalations around 2–3.5 Å.^[^ ^42^ ^]^ In addition, the oxidized Pt‐O peaks were identified at around ≈1.75 Å.^[^ ^43^ ^]^ Further EXAFS oscillations in k space (k^2^‐weighting) of PtNDs@CoS_2_‐NrGO and Pt‐foil for Pt‐L_3_ edge oscillation frequencies can be found in Figure S8 (Supporting Information). XANES and EXAFS responses for Co in PtNDs@CoS_2_‐NrGO, CoS_2_‐NrGO and Co‐foil are provided in Figure 3h,i. Their corresponding K‐edge EXAFS oscillation frequencies in k space (k^2^‐weighting) are shown in Figure S9 (Supporting Information). These findings are consistent with the electronic and elemental composition investigation conducted using XPS responses of Co and S elements.

### 2.2 Electrocatalytic Activity for Various Alcohols

The electrochemical activity of typically prepared PtNDs@CoS_2_‐NrGO, Pt‐NrGO and commercial Pt‐C electrocatalysts was measured in an acidic environment using cyclic voltammetry in the region of ‐0.2 to 1.2 V versus Ag/AgCl at a scan rate of 50 mV s^–1^ in a 0.5 m H_2_SO_4_ solution. This is the region where hydrogen adsorption and desorption on Pt take place. To determine the number of active catalytic sites available for PtNDs@CoS_2_‐NrGO, Pt‐NrGO, and commercial Pt‐C electrocatalysts, electrochemical active surface area (ECSA) calculations were performed in the backward sweep of the CV curve from the range of −0.184 to 0.128 V as shown in Figure S10a (Supporting Information). The ECSA of the electrocatalysts was calculated using the following conventional equations^[^ ^44^ ^]^

(1) $$\mathrm{Charge}\;\left[C\right]=\int_{t0}^{t1}Idt=\int_{-0.184}^{0.128}\frac{I\left[A\right]\times dE\left[V\right]}{v\left[\frac{V}{s}\right]}\;$$

(2) $$\mathrm{ECSA}\left[cm^{2}\;\mathrm{pt}\right]\;=\;\frac{\mathrm{charge}\;\left[\mu C\right]}{Q\;\left[\frac{\mu C}{\;cm^{2}\mathrm{pt}}\right]}$$

where *C* is the average charge integrated from hydrogen adsorption/desorption, *I* is the current, *Q* is the charge collection of hydrogen adsorption/desorption *Q*_H_ after the double layer correction (the charge needs to oxidize the single layer Pt surface and assumed to be *Q*_ref_ = 210 µC cm^–2^), and *v* is the scan rate.

The PtNDs@CoS_2_‐NrGO electrocatalyst showed a higher ECSA value of 42.86 m^2^g^–1^ as compared to Pt‐NrGO (29.95 m^2^g^–1^) and Pt‐C (23.15 m^2^g^–1^) in Figure S10b (Supporting Information).^[^ ^8^ ^]^ The reduction peak of PtNDs@CoS_2_‐NrGO shifted more negatively compared to the commercial Pt‐C electrocatalyst due to the successful formation of PtNDs on the CoS_2_‐NrGO.^[^ ^8^, ^45^ ^]^ Such improved electrocatalytic activity of PtNDs@CoS_2_‐NrGO is due to their enlarged active specific surface area and high‐index exposed crystal facets, which could allow excellent transport of molecules in the catalyst medium. Additionally, dendritic structures facilitate high electrocatalytic activity due to rich catalytically active edges and corners of structures.^[^ ^45^ ^]^ This providing sufficient catalytic sites and adequate absorption sites for alcohol molecules during the electrochemical performance.

### 2.3 Electrochemical Studies of Methanol Oxidation

Anode and cathode electrocatalysts play an important role in improving the efficiency of DMFC and the overall DMFC cell reaction in Equations (3)–(5)^[^ ^46^ ^]^

(3) $$CH_{3}\mathrm{OH}+H_{2}O\;\to 6H+6e+CO_{2}\;\left(\text{Anode}\;\text{reaction}\right)$$

(4) $$O_{2}+4H+4e\;\to 2H_{2}O\;\left(\text{Cathode}\;\text{reaction}\right)$$

(5) $$2CH_{3}\mathrm{OH}+3O_{2}\to 2CO_{2}+4H_{2}O\;\left(\text{Overall}\;\text{reaction}\right)$$

**Figure 4:**
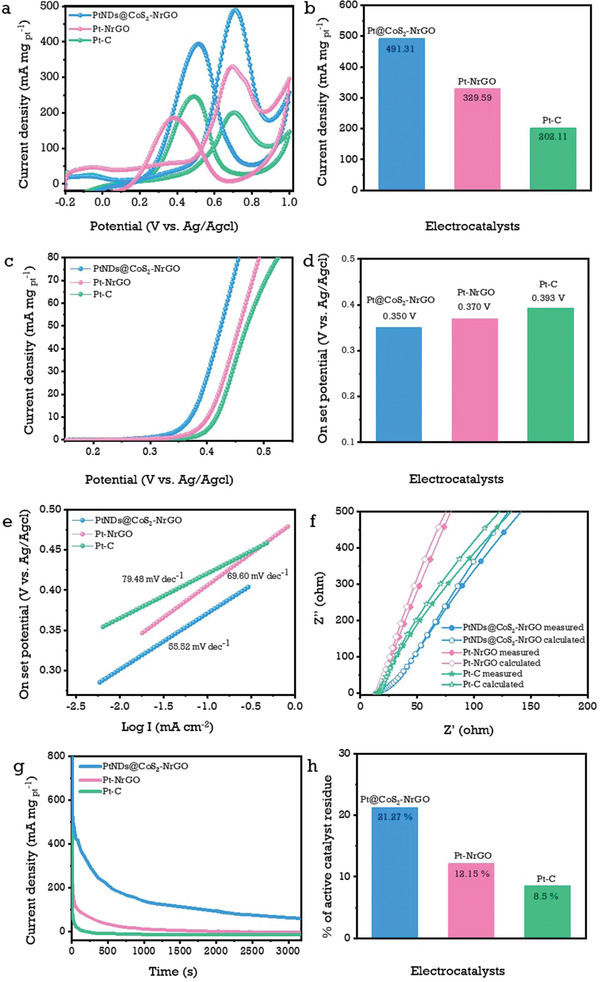
Representative methanol oxidation studies and their characteristic curves: a,b) mass activities polarization curves and their corresponding bar chart values; c,d) linear sweep voltammetry and corresponding onset‐potential bar chart values; e) parallel Tafel slope curves; f) EIS curves with the calculated electrical resistances of 14.73, 16.73, and 15.51 Ω using the Randles circuit fitting method; g,h) chronoamperometric responses and their corresponding bar chart values of PtNDs@CoS_2_‐NrGO, Pt‐NrGO, and commercial Pt‐C electrocatalysts, respectively in 1 m MeOH + 0.5 m H_2_SO_4_ electrolyte solutions.

**Figure** **4** shows the basic electrochemical exploration profiles for the methanol oxidation reaction (MOR) to PtNDs@CoS_2_‐NrGO, Pt‐NrGO and commercial Pt‐C electrocatalysts in a 0.5 m H_2_SO_4_ acidic medium. Figure 4a shows the CV curves at a sweep rate of 50 mV s^–1^, which consists of two sharp peaks in the forward and backward scans. During the oxidation of MeOH molecules, a clear sharp forward peak appeared at 0.68 V. Subsequently, the reaction intermediates were obtained on the catalyst surface. During the backward scan, the reactive carbonaceous intermediates were converted into CO_2_ through further oxidation with water molecules, which can regenerate active metal‐Pt sites and allow for longer oxidation of methanol molecules on the catalyst surface.^[^ ^47^ ^]^ As shown in Figure 4a, the synthesized PtNDs@CoS_2_‐NrGO exhibit a higher oxidation current density of 491.31 mA mg_pt_^–1^ compared to Pt‐NrGO and Pt‐C, which exhibit 329.59 mA mg^–1^_Pt_ and 202.11 mA mg_pt_^–1^, respectively. Table S6 (Supporting Information) shows the PtNDs@CoS_2_‐NrGO electrocatalyst shows excellent ECSA and methanol oxidation current density as compared to recently reported literature.

Besides that, the LSV responses in Figure 4c reveal that the onset potential of PtNDs@CoS_2_‐NrGO (0.35 V) was lower than Pt‐NrGO (0.37 V) and Pt‐C (0.39 V), respectively. This effect might be due to the oxygenation support of cobalt atoms in PtNDs@CoS_2_‐NrGO.^[^ ^48^ ^]^ However, the faster kinetics of methanol molecules were observed without further hindrance and the corresponding calculated Tafel slopes are presented in Figure 4e. The lowest Tafel slope was 55.52 mV dec^–1^ obtained for PtNDs@CoS_2_‐NrGO.^[^ ^15^ ^]^ On the other hand, Pt‐NrGO and Pt‐C showed values of 69.60 and 79.48 mV dec^–1^, respectively. Figure 4f shows Nyquist plots for the electrochemical resistance evaluations of PtNDs@CoS_2_‐NrGO, Pt‐NrGO, and Pt‐C in 1 m MeOH and 0.5 m H_2_SO_4_, where the obtained curves were fitted using the Randles method and exhibit values of 14.73, 16.73, and 15.51 Ω, respectively. PtNDs@CoS_2_‐NrGO electrocatalyst exhibits a lower resistivity against methanol molecules due to the higher conductivity of cobalt sulfide and graphene.^[^ ^15^ ^]^ In Figure 4g, the durability measurements for PtNDs@CoS_2_‐NrGO, Pt‐NrGO, and commercial Pt‐C in chronoamperometry at 0.65 V in 1 m MeOH and 0.5 m H_2_SO_4_ for 3000 s were presented. The long‐term cycling performance of the PtNDs@CoS_2_‐NrGO electrocatalyst has been conducted for 500 cycles at a sweep a rate of 50 mV s^–1^ in 1 m MeOH and 0.5 m H_2_SO_4_ as shown in Figure S12 (Supporting Information). The results indicate that the current density ratio of forward and backward scan (*J*_f_/*J*_b_) was decreased gradually from 1.13 to 0.918 after 500 CV cycles by adsorbing the CO intermediates strongly on Pt surfaces and current density was decreased to 459.02 mA mg_pt_^–1^ from 491.23 mA mg_pt_^–1^. On the other hand, the current density of PtNDs@CoS_2_‐NrGO was reduced to 459.02 mA mg_pt_^–1^ (stable for 93.4%) from its initial current density of 491.23 mA mg_pt_^–1^ after 500 cycles of CV, which demonstrates the excellent cycling durability of PtNDs@CoS_2_‐NrGO.

Moreover, we investigated the changes in morphology after the long‐term CV cycling stability and long‐term durability test using TEM and XRD analysis as shown in Figures S13 and S14 (Supporting Information). A detailed discussion is given in the supporting information.

### 2.4 Electrochemical Studies of Ethylene Glycol (EG) Oxidation

**Figure 5:**
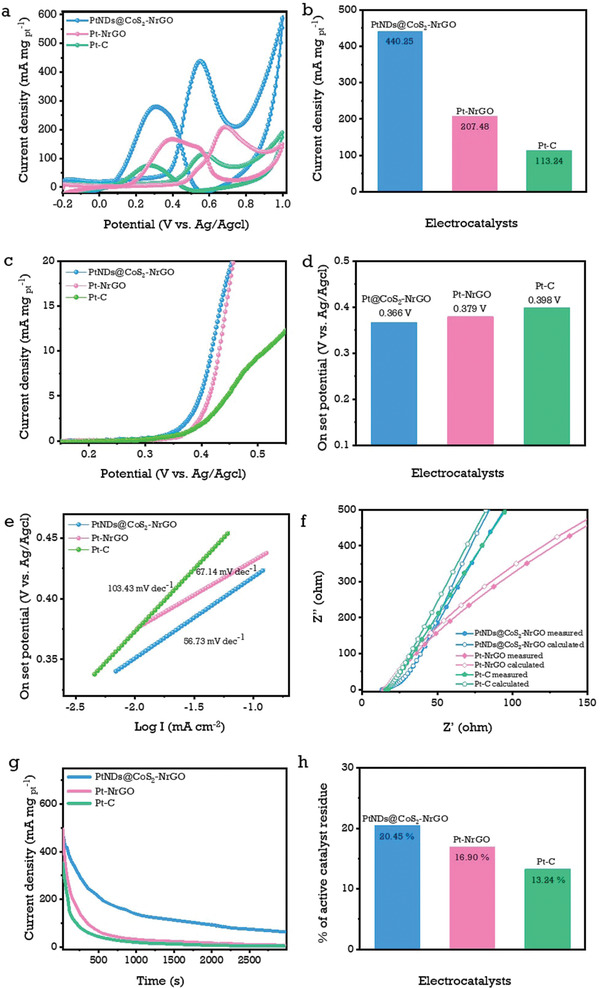
Representative ethylene glycol oxidation studies and their characteristic curves: a,b) mass activities polarization curves and their corresponding bar chart values; c,d) linear sweep voltammetry and their corresponding onset‐potential bar chart values; e) parallel Tafel slope curves; f) EIS curves with the calculated electrical resistances of 14.56, 15.56, and 17.23 Ω using the Randles circuit fitting method; g,h) chronoamperometric responses and their corresponding bar chart values of PtNDs@CoS_2_‐NrGO, Pt‐NrGO, and commercial Pt‐C electrocatalysts in 1 m ethylene glycol + 0.5 m H_2_SO_4_ electrolyte solutions.

In contrast to the methanol oxidation reaction, the ethylene glycol oxidation reaction (EGOR) necessitates multiple steps of reactions to achieve the desired CO_2_. Further investigation into the electrochemical activity of ethylene glycol was evaluated using the PtNDs@CoS_2_‐NrGO, Pt‐NrGO, and commercial Pt‐C electrocatalyst against 1 m ethylene glycol in 0.5 m H_2_SO_4_ solution. In **Figure** **5**a, the CV polarization curves show the better current density of 440.25 mA mg_Pt_^–1^ for the PtNDs@CoS_2_‐NrGO electrocatalyst, which is 2.12 and 3.86 times higher than the Pt‐NrGO and commercial Pt‐C electrocatalyst, respectively. Table S7 (Supporting Information) shows the PtNDs@CoS_2_‐NrGO electrocatalyst shows excellent EG electro‐oxidation activity as compared to recently reported literature.

Due to the multiple scission requirements of C—H, C—C, and C—O bonds in EGOR, the mass activity values are lower than MOR. Furthermore, the LSV evaluation reveals that the PtNDs@CoS_2_‐NrGO electrocatalyst exhibit the lowest onset potential of 0.366 mV than Pt‐NrGO and commercial Pt‐C electrocatalyst, as shown in Figure 5c,d. In line with the Sabatier principle, the interactions between the metal sulfides and OH adsorption species are neither too strong nor too weak.^[^ ^10^ ^]^ This facilitates the Pt sites to achieve better reaction kinetics of EG oxidation. According to the derived Tafel slope, PtNDs@CoS_2_‐NrGO is 56.73 mV dec^–1,^ for Pt‐NrGO and Pt‐C are 67.14 and 103.43 mV dec^–1^, respectively (Figure 5e). As a result, the smaller Tafel slope of PtNDs@CoS_2_‐NrGO implies faster‐reaction kinetics.^[^ ^49^ ^]^

Electrochemical impedance spectroscopy (EIS) was measured with 1 m EGOH in 0.5 m H_2_SO_4_ as shown in Figure 5f. Furthermore, the obtained curves were fitted using the Randles model (Figure 5f). This charge transfer capability can further support the faster reaction kinetics.^[^ ^49^ ^]^ The stability of electrocatalysts was examined by a chronoamperometric test. Figure 5g displays the highest current density after 1100 s of the chronoamperometric test, where PtNDs@CoS_2_‐NrGO shows 72.5 mA mg_pt_^–1^, which is 4.83 and 8.14 times higher than Pt‐NrGO and commercial Pt‐C electrodes, respectively.^[^ ^15^ ^]^ Long‐term CV cycling tests were also conducted in Figure S15 (Supporting Information), which revealed that PtNDs@CoS_2_‐NrGO electrocatalyst capability of high retentivity after 500 CV cycle tests. The current density ratio of J_f_/J_b_ was decreased gradually from 1.3 to 1.0 after 500 cycles of CV, which demonstrates low impairment of CO tolerance of PtNDs@CoS_2_‐NrGO. Notably, the current density of PtNDs@CoS_2_‐NrGO was reduced to 372.02 mA mg_pt_^–1^ (stable for 85.9%) from its initial current density of 433.23 mA mg_pt_^–1^ after 500 cycles of CV, demonstrating good cycling durability. After 500 CV cycles of PtNDs@CoS_2_‐NrGO electrocatalyst, we investigated the morphology changes and chemical composition of the catalysts by using TEM analysis as shown in Figure S16 (Supporting Information). Also, the post morphology analysis of long‐term stability was evaluated using XRD analysis in Figure S17 (Supporting Information). Further, detailed discussion was given in the supporting information.

### 2.5 Electrochemical Studies of Glycerol Oxidation

**Figure 6:**
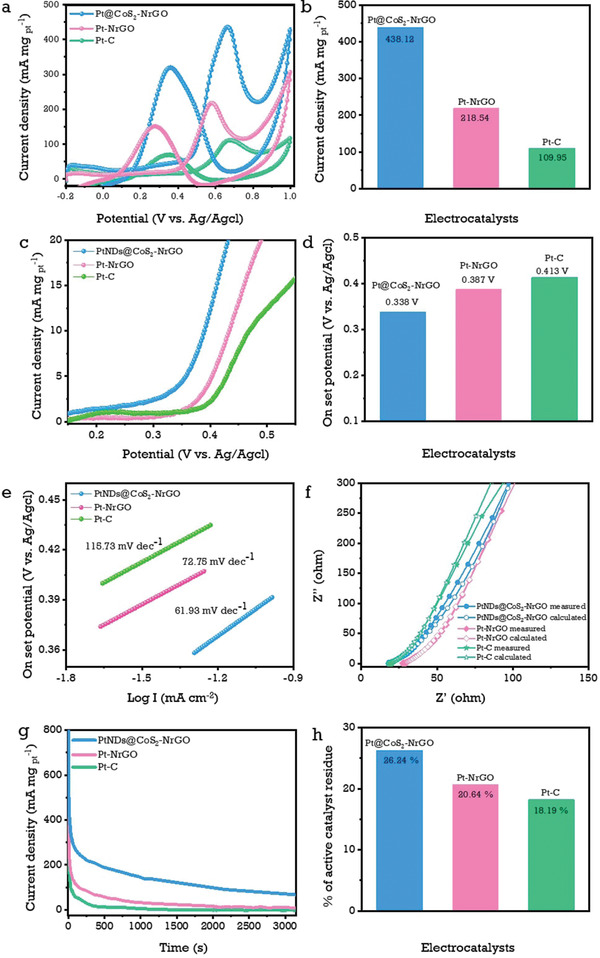
Representative glycerol oxidation studies and their characteristic curves: a,b) mass activities polarization curves and their corresponding bar chart values; c,d) linear sweep voltammetry and their corresponding onset‐potential bar chart values; e) parallel Tafel slope curves; f) EIS curves with the calculated electrical resistance of 15.16, 17.36, and 16.79 Ω using the Randles circuit fitting method; g,h) chronoamperometric responses and their corresponding bar chart values of PtNDs@CoS_2_‐NrGO, Pt‐NrGO, and commercial Pt‐C electrocatalysts in 1 m glycerol + 0.5 m H_2_SO_4_ electrolyte solutions.

Glycerol is one of the most crucial polyhydric C3 type alcohols in nature and glycerol electro‐oxidation performance was investigated by using prepared PtNDs@CoS_2_‐NrGO electrocatalyst were evaluated in 1 m glycerol in 0.5 m H_2_SO_4_ solution. The CV results in **Figure** **6**a shows that the PtNDs@CoS_2_‐NrGO electrocatalyst possesses a high current density of 438.12 mA mg_Pt_^–1^_,_ which is 2.0 and 3.98 times higher than Pt‐NrGO and commercial Pt‐C. Table S8 (Supporting Information) compares the ECSA and glycerol oxidation current densities obtained with the PtNDs@CoS_2_‐NrGO electrocatalyst to previously reported literature. During the oxidation process, glycerol might undergo various types of complex oxidation reactions on the PtNDs@CoS_2_‐NrGO electrocatalysts in an acidic medium and produce an undesired intermediate, namely glyceraldehyde, formic acid, and glycolic acid^[^ ^50^, ^51^ ^]^

(6) $$\begin{array}{ccc} CH_{2\;}\mathrm{OH} & - & \mathrm{CHOH}-CH_{2\;}\mathrm{OH}\;+\;20\;OH^{-}\;\to \;3{\mathrm{CO}}_{3}^{2-}\;\\ & & +\;14\;H_{2}O\;+14e^{-} \end{array}$$

Gomes et al. reported the formation of the intermediates during the glycerol electro‐oxidation on the Pt catalyst by in situ FTIR experiments.^[^ ^52^ ^]^ During the electro‐oxidation of glycerol, formic acid is a major intermediate formed on the catalyst surface and it led to the formation of poisonous CO_ads_ on Pt‐active sites on the catalyst surface. The CoS_2_ and NrGO can generated enough amount of OH species in the removal of poisons intermediate of CO_ads_ on Pt‐active sites.^[^ ^31^, ^53^ ^]^ Therefore, PtNDs@CoS_2_‐NrGO catalyst confirmed their superior electrocatalytic activity toward the alcohol fuels. Figure 6c,d shows LSV polarization in which the onset potential values followed the trend of PtNDs@CoS_2_‐NrGO < Pt‐NrGO < Pt‐C and their derivative Tafel slopes were about 61.93, 72.75, and 115.73 mV dec^–1,^ respectively.^[^ ^13^ ^]^ The charge transfer capacity of PtNDs@CoS_2_‐NrGO, Pt‐NrGO, and commercial Pt‐C are depicted in Figure 6f as Nyquist plots, along with their respective charge resistances of 15.16, 17.36, and 16.79 Ω measured using the Randles fitting method. PtNDs@CoS_2_‐NrGO exhibits lower charge resistance due to the high conducting carbon support and allows for faster mobilization of charge carriers.^[^ ^51^ ^]^ In the chronoamperometric test, PtNDs@CoS_2_‐NrGO electrodes degrade at a slower rate at 1–1200 s of operation. As shown in Figure 6g,f, after attaining the thermodynamic quasiequilibrium steady state, the retentivity % of PtNDs@CoS_2_‐NrGO is 1.27 and 1.44 times higher than that of Pt‐NrGO and commercial Pt‐C electrodes, respectively. The long‐term cycling stability tests for glycerol oxidation are shown in Figure S18 (Supporting Information). After 500 CV cycles, the current density ratio *J*_f_/*J*_b_ for the PtNDs@CoS_2_‐NrGO electrocatalyst was reduced to 1.04 from its initial value of 1.22. However, after 500 cycles of CV, the current density of PtNDs@CoS_2_‐NrGO was reduced to 408.14 mA mg_pt_^–1^ (stable for 92.5%) from its initial current density of 441.08 mA mg_pt_^–1^. The lowering in the current density occurs due to the stronger adsorption of CO intermediate on Pt surface.^[^ ^54^ ^]^ This demonstrates the high cycle endurance of PtNDs@CoS_2_‐NrGO. We also investigated the morphology changes and chemical composition of PtNDs@CoS_2_‐NrGO electrocatalyst after 500 CV cycles using TEM and XRD analysis, as shown in Figures S19 and S20 (Supporting Information). In addition, a detailed discussion was provided in the supporting information.

### 2.6 Mechanism of PtNDs@CoS_2_‐NrGO Catalyst toward Electro‐Oxidation of Alcohols

The aforementioned results confirm that PtNDs@CoS_2_‐NrGO shows improved electro‐oxidation of methanol, ethylene glycol and glycerol with high oxidation current density, lower onset peak potential, and smaller Tafel slope when compared to other prepared catalysts such as Pt‐NrGO and Pt‐C catalyst. Poisonous intermediates (Pt‐(CO)_ads_) were formed during the electro‐oxidation of alcohol molecules on the PtNDs@CoS_2_‐NrGO catalyst.^[^ ^55^ ^]^ Similarly, the formation of formic acid is a major by‐product during ethylene glycol^[^ ^51^, ^54^, ^56^ ^]^ and glycerol electro‐oxidation.^[^ ^51^ ^]^ The formation of poisonous CO_ads_ on the Pt‐active sites of the catalyst surface further hinders the catalytic activity. In order to reactivate the Pt active sites on the catalyst, the accumulated CO_ad_ species must be removed from the surface of the PtNDs@CoS_2_‐NrGO catalyst. The adsorbed OH species promotes the oxidation of CO to CO_2_.^[^ ^31^, ^53^, ^56^ ^]^

The exposed Co and S sites in the CoS_2_ structure, as well as the C—N defected N‐doped reduced graphene oxide, are better at activating water molecules and generating the abundant active OH species. The generated OH species oxidizes the CO_ads_ into CO_2_ and regenerates the Pt active sites for further oxidation of alcohols.^[^ ^31^, ^53^, ^56^ ^]^ As a result, our results demonstrate that PtNDs@CoS_2_‐NrGO has a superior electrocatalytic activity for better electro‐oxidation of alcohols

(7) $$\left.\begin{array}{c} \mathrm{HOC}H_{2}\mathrm{CH}\left(\mathrm{OH}\right)CH_{2}\mathrm{OH}\\ \mathrm{HOC}H_{2\;}CH_{2}\mathrm{OH}\;\\ CH_{3\;}\mathrm{OH} \end{array}\right\}+\mathrm{PtNDs}@\mathrm{Co}S_{2\;}-\mathrm{NrGO}\to \mathrm{PtNDs}@\mathrm{Co}S_{2\;}-\mathrm{NrGO}-CO_{\mathrm{ad}\;}+xH^{+}+xe^{-}$$

(8) $$\mathrm{PtNDs}@\mathrm{Co}S_{2\;}-\mathrm{NrGO}+H_{2}O\to \mathrm{PtNDs}@\mathrm{Co}S_{2\;}-\mathrm{NrGO}-OH_{\mathrm{ads}}+H^{+\;}+e^{-}$$

(9) $$\mathrm{PtNDs}@\mathrm{Co}S_{2\;}-\mathrm{NrGO}-CO_{\mathrm{ad}\;}+\;\text{PtNDs}@\mathrm{Co}S_{2\;}-\mathrm{NrGO}-OH_{\mathrm{ads}}\;\to \;\text{PtNDs}@\mathrm{Co}S_{2\;}-\mathrm{NrGO}+\;CO_{2}+H^{+}\;+e^{-}$$

### 2.7 Computational Methodology and Results

**Figure 7:**
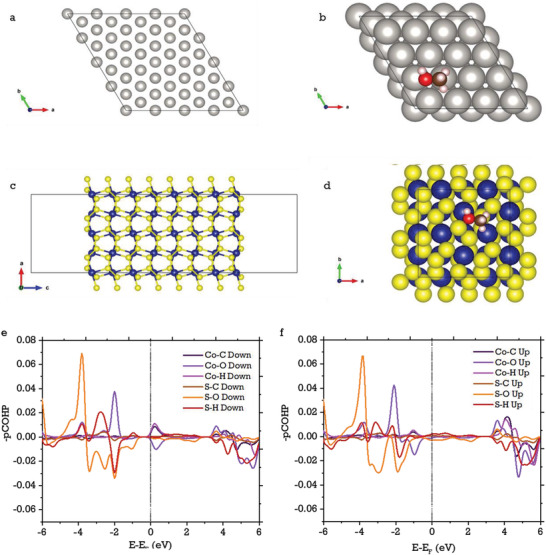
a) The surface slab of Pt (111), b) methanol molecule adsorbed on Pt (111) surface, c) surface slab of CoS_2_ (200), and d) methanol molecule adsorbed on CoS_2_ (200) surface, e,f) projected COHP (‐pCOHP) plots of methanol adsorbed CoS_2_ (200) surface with different atomic interactions in up and down spin channels.

To understand the synergistic effects between the metal surfaces and alcohol molecules, computational density functional theory (DFT) calculations were carried out within the Vienna ab initio simulation package (VASP). The detailed computational methodology can be found in the supporting information. The relaxed bulk structures were used in obtaining the surface slabs of Pt (111) and CoS_2_ (200) and in our calculations. We opted for CoS_2_ (200) surfaces to adsorb methanol molecule as shown in **Figure** **7**a–d. The reason behind this choice is given in the discussion section. Prior to the methanol adsorption, the total energy of a free methanol molecule in a 15 × 15 × 15 Å cubic box was calculated by relaxing the ionic positions alone. Considering the computational cost of surface and adsorption calculations, we selected the low‐indexed Pt (111) and CoS_2_ (200) surfaces that are observed in HR‐TEM micrographs. Our modelled Pt (111) surface slab has 9 atomic layers with a thickness of around 18.4 Å, where each layer has 16 Pt atoms and hence, a total of 144 atoms are present in the surface slab. Similarly, the CoS_2_ (200) surface slab has a thickness of around 20.7 Å but has only 8 atomic layers and a total of 192 atoms (64 Co and 128 S atoms) are present in the surface slab. A vacuum spacing of 15 Å was applied by keeping the surface slab at the center, where both the top and bottom layers were exposed to vacuum evenly, in addition to the applied dipole corrections perpendicular to the surface slabs. For the surface slab relaxation, only the top and bottom two layers were relaxed, and the middle layers were assumed to be bulk. Following the relaxation of surface slabs, the methanol adsorbed surface relaxation was carried out and the results are presented.

The surface energy, *γ*, was calculated using the formula,

(10) $$\gamma \;=\frac{E_{\mathrm{surface}}-nE_{\mathrm{bulk}}}{2A}$$

In Equation (3), *E*_surface_ and *E*_bulk_ are the total energies of the relaxed surface slab and bulk unit cell, *n* is the number of bulk unit cells, and *A* is the surface area of the slab. In general, the surface energy determines the surface stability, and it is simply an extra energy exhibited by the atoms present at the surface.

The calculated surface energy for Pt (111) is 1.45 J/m^2^, which is in close agreement with the previous DFT‐PBE calculation.^[^ ^57^ ^]^ Meanwhile, the calculated surface energy for CoS_2_ (200) is 0.47 J m^−2^, which is much lower than the Pt (111) surface as shown in Table S7 (Supporting Information). This means that the CoS_2_ (200) surface is thermodynamically more favorable than the Pt (111) surface. Therefore, the surface of CoS_2_ (200) is more readily available for methanol adsorption compared to Pt (111), where the catalytic reaction occurs, and we also calculated the adsorption energy, *E*_ads_ as follows

(11) $$E_{\mathrm{ads}}=E_{\mathrm{surface}+\mathrm{methanol}}\;-E_{\mathrm{surface}}-E_{\mathrm{methanol}}$$

Here, *E*_surface + methanol_ is the total energy of methanol adsorbed on the CoS_2_ (200) surface slab, *E*_surface_ is the total energy of the pure surface without adsorption of methanol, and *E*_methanol_ is the total energy of free methanol molecule calculated in a 15 × 15 × 15 Å cubic box, respectively. The adsorption energy of methanol over the Pt (111) surface has already been determined recently^[^ ^58^ ^]^ through DFT calculations (using the same VASP code), and it is calculated to be −0.49 eV. Therefore, we have omitted this step in our calculation. However, we have calculated the adsorption energy of methanol over a CoS_2_ (200) surface and it is calculated to be −0.14 eV as shown in Table S9 (Supporting Information). When the adsorption energies of methanol over Pt (111) and CoS_2_ (200) surfaces are examined, the values are found to be relatively low, signifying that the adsorption is feasible on both surfaces, as indicated by their near‐zero negative values. However, the desorption of methanol is slightly easier on the CoS_2_ (200) surface than on the Pt (111) surface, possibly due to the weakened adsorption, which may activate the cleavage of intermediate species during the catalytic oxidation reaction.

To gain a better understanding of the adsorption/desorption behavior of methanol at the CoS_2_ (200) surface, we have calculated the pCOHP and ICOHP using the LOBSTER code.^[^ ^59^ ^]^ The pCOHP analysis can be used to determine the bond strength of adsorbed species on a surface. The Fermi level is set to zero for this analysis. The pCOHP values are typically plotted as ‐pCOHP values, and we used the same methodology to determine the pCOHP of the methanol adsorbed CoS_2_ surface (200), where positive values of ‐pCOHP represent bonding interactions, and negative values of ‐pCOHP represent antibonding interactions.^[^ ^60^ ^]^ The ‐pCOHP interactions are plotted for both up and down spin channels as shown in Figure 7e,f, and the integrated COHP (ICOHP) values of corresponding interactions are listed in Table S10 (Supporting Information). The bonding/antibonding interactions between Co—C (carbon atom of methanol), Co—O (oxygen atom of methanol), and Co—H (hydrogen atom of the C—H linkage of methanol) were calculated. Similarly, S—C, S—O, and S—H interactions were also calculated, since the surface slab of CoS_2_ (200) is terminated by both Co and S atoms present in the top layer of the surface. The results reveal that the stronger bonding interactions arise from the Co—O and S—O bonds. However, significant antibonding interactions were found for S—O bonds in the valence band. As a result, the oxygen atoms in the methanol molecule preferentially bind to the Co atoms on the surface slab. Whenever antibonding states are present near the Fermi level, structural instability occurs. A small number of antibonding states of Co—O are present near the Fermi level in the valence band of the up‐spin channel and near the Fermi level in the conduction band of the down spin channel, indicating that Co—O bond breaking is feasible. The bonding interactions of Co—C occur at the vicinity of the Fermi level in the conduction band region of the down spin channel. A weak S—H (hydrogen bonding with sulfur) is also revealed from the calculated ICOHP values. The ICOHP values that reflect the strength of bonds support our discussion of ‐pCOHP results. The DFT calculations yield the following conclusions that corroborate the experimental results: i) The surface energy of CoS_2_ (200) is significantly lower than Pt (111) and therefore the surface contact of CoS_2_ (200) in the PtNDs@CoS_2_‐NrGO composite is readily available for adsorption of methanol. ii) While adsorption of methanol is more feasible on a Pt (111) surface than on a CoS_2_ (200) surface, the relatively small negative adsorption energy of the CoS_2_ (200) surface facilitates the desorption of the methanol molecule for further oxidation steps. iii) The statement (ii) is supported by ‐pCOHP results, which show that a small number of antibonding states of Co—O near the Fermi level represent the structural instability in addition to significant bonding contributions.

## 3 Conclusion

In summary, we successfully fabricated quasihexagonal structured PtNDs cages on CoS_2_‐NrGO using an engineering‐shape‐controlled and size‐orientation strategy through an agile one‐pot hydrothermal method followed by a simple chemical reflux method. Physical and surface morphology characterizations of PtNDs@CoS_2_‐NrGOs were extensively scrutinized using XRD, Raman, XPS, XAS, AFM, HR‐TEM, and FE‐SEM to successfully form PtNDs on CoS_2_ surfaces. The growth of PtNDs on the CoS_2_‐NrGO architecture provided an enhanced electrochemical oxidation of methanol, ethylene glycol and glycerol in 0.5 m H_2_SO_4_ acidic medium and showed peak current densities of 491.31, 440.25, and 438.12 mA mg_pt_^–1^, respectively. The CoS_2_ and C—N defected N‐doped reduced graphene oxide improve the production of active OH species on the catalyst surface, resulting in the recreation of Pt‐active sites from poisons of Pt‐CO_ad_ during alcohol oxidation, as well as improved durability. Our DFT calculations revealed that the surface energy of CoS_2_ (200) is significantly lower than that of Pt (111); thus, the surface contact of CoS_2_ (200) in PtNDs@CoS_2_‐NrGO can readily adsorb methanol. A relatively small negative adsorption energy of the CoS_2_ (200) surface facilitates the desorption of methanol molecule for further oxidation steps. This observation is also consistent with the ‐pCOHP results, where a small number of antibonding states of Co—O near the Fermi level indicates certain structural instability in addition to significant bonding contributions. Additionally, postmortem analysis after 500 CV cycles showed better catalytic morphology and material retention capacity. The presented material interaction studies create a new path for the effective utilization of precious materials and highlight the efficient material fabrication techniques for future electrochemical applications.

## 4 Experimental Section

### Materials and Chemicals

All purchased chemicals were analytical standards and they were used without being purified further. Cobalt chloride (II) (CoCl_2_, 98%), potassium tetrachloroplatinate (II) (K_2_PtCl_4_, 99.5%), natural graphite powder, potassium permanganate (KMnO_4_, 99.3%), thiourea (CH_4_N_2_S, 99%), glycerol (C_3_H_8_O_3_, 99%), commercial Pt‐C (20% Pt‐C), and ethylene glycol (C_2_H_6_O_2_, 99%) were procured from Alfa Aesar. Ascorbic acid (AA) (C_6_H_8_O_6,_ 99%), hydrochloric acid (HCl, 35%), hydrogen peroxide (H_2_O_2_, 32%), and sulfuric acid (H_2_SO_4_, 95%) were ordered from Daejung Chemicals. In addition, a 5  wt% Nafion solution, Pluronic F‐127, and methanol were obtained from Sigma‐Aldrich and Samchun Pure Chemical Co., LTD. (South Korea), respectively.

### Synthesis of CoS_2_‐NrGO

Highly exfoliated GO was synthesized using natural graphite powder through a modified Hummer's route.^[^ ^61^ ^]^ An appropriate amount of obtained GO powder was dispersed in 30 mL of deionized (DI) water and ultrasonicated for 3 h, which yielded few‐layered graphene oxides. Then, a mixture of 7 × 10^−3^ m CoCl_2_ and 14 × 10^−3^ m thiourea were prepared using 15 mL of DI water separately, which underwent mechanical stirring for 2 h. The above solution was gradually added to the dispersed GO solution and stirred for another 3 h. Next, the mixture was transferred to a 100 mL Teflon‐lined autoclave and maintained at 180 °C for 24 h in an electric oven. The obtained product was cooled down to room temperature and then washed several times with DI water and ethanol. Finally, the retrieved product was dried at 65 °C under vacuum overnight.

### Synthesis of Platinum Nanodendrites@CoS_2_‐NrGO

Platinum nanodendrites (PtNDs) were homogeneously decorated on the CoS_2_‐NrGO surfaces using a wet chemical reflux method. An appropriate amount of CoS_2_‐NrGO (1 mg mL^–1^) was dispersed into 30 mL of DI water and stirred for 1 h. Then, 24 × 10^−3^ m of K_2_PtCl_4_ and F‐127 (30 mg) were dissolved in 7.5 mL of DI water and gently added to the CoS_2_‐NrGO solution, followed by 3 h of stirring. Following that, 0.1 m of environment‐friendly ascorbic acid (AA) in 7.5 mL of DI water was gradually added to the above mixture, which was maintained at 95 °C under reflux conditions for 18 h. Finally, the resultant product was rinsed with water and ethanol until it attained a neutral pH. It was then dried in a vacuum oven for 24 h at 65 °C. For comparison, Pt@NrGO was also synthesized using a similar synthesis procedure without a cobalt source.

### Electrochemical Evaluation and Characterization

Appropriate weights of typically obtained PtNDs@CoS_2_‐NrGO, Pt‐NrGO, and commercial Pt‐C were dispersed in a mixture of 0.4 mL ethanol/water mixture (v/v ratio 2:1) with 10 µL of a 5 wt% Nafion solution. The resultant mixture was ultrasonicated for 30 min to obtain a homogeneously dispersed catalyst ink. A conventional three‐electrode cell set‐up consists of a modified glassy carbon electrode (GCE) which was polished using 1 and 0.3 µm alumina slurries in sequence, followed by washing with DI water and used as a working electrode. Pt‐wire and Ag/AgCl electrode with saturated KCl were used as counter and reference electrodes, respectively. 5 µL of homogeneously dispersed catalyst ink containing 1.0 mg_Pt_ mL^–1^ was dripped on the GCE and dried at room temperature. The electrochemical active surface area was calculated by sweeping the modified working electrode in the potential range of −0.2 to 1.2 V versus SCE in a 0.5 m H_2_SO_4_ analyte solution. To evaluate the electrocatalytic activities of C1, C2, and C3 alcohols in acidic conditions, cyclic voltammetry was conducted in 0.5 m H_2_SO_4 _+ 1 m methanol, 0.5 m H_2_SO_4 _+ 1 m ethylene glycol, and 0.5 m H_2_SO_4 _+ 1 m glycerol, at scan rates of 50 mV s^–1^. Furthermore, the cyclic stability and durability properties of synthesized electrocatalysts were examined using long‐term CV tests and chronoamperometric analysis at each catalyst peak current density. For better comparisons, Pt‐NrGO and commercial Pt‐C electrocatalytic activities were also evaluated using the same procedure. Electrochemical impedance spectroscopy was also performed on all the corresponding electrolytes, followed by analysis using the standard Randles circuit fitting method. To obtain the post morphology XRD evaluations for all the three electrolytes, an appropriate amount of catalyst was dissolved in 0.4 mL ethanol/water mixture (v/v ratio 2:1) and sonicated for a certain period and coated on the (1 cm × 1 cm) carbon paper and dried in an ambient condition. All the electrochemical potentiostat evaluations were carried out using a Gamry instrument (Reference 600/ZRA) at ambient conditions.

## Conflict of Interest

The authors declare no conflict of interest.

## Supporting information

Supporting Information

## Data Availability

Research data are not shared.
