# Supplementary material for: Quasihexagonal Platinum Nanodendrites Decorated over CoS2‐N‐Doped Reduced Graphene Oxide for Electro‐Oxidation of C1‐, C2‐, and C3‐Type Alcohols
Source: Adv Sci (Weinh). 2022 Jan 20;9(8):2105344. doi: 10.1002/advs.202105344 (PMC8922112; doi:10.1002/advs.202105344)
Supplement: Supplementary file 1 — Supporting Information [file ADVS-9-2105344-s001.pdf]

ADVANCED SCIENCE
Open Access

Supporting Information

for *Adv. Sci.,* DOI: 10.1002/advs.202105344

Quasi-hexagonal platinum nanodendrites decorated over CoS_2-N doped reduced graphene oxide for electrooxidation of C1, C2 and C3-type alcohols

*Natarajan Logeshwaran, Iyyappa Rajan Panneerselvam, Shanmugam Ramakrishnan, Ramasamy Santhosh Kumar, Ae Rhan Kim, Yan Wang, Dong Jin Yoo**

Supporting Information

Quasi-hexagonal platinum nanodendrites decorated over CoS_2-N doped reduced graphene oxide for electrooxidation of C1, C2 and C3-type alcohols

Natarajan Logeshwaran [a], Iyyappa Rajan Panneerselvam [b], Shanmugam Ramakrishnan [a], Ramasamy Santhosh Kumar [a], Ae Rhan Kim [a, c], Yan Wang [b], Dong Jin Yoo[a, c]*

Natarajan Logeshwaran [a], Shanmugam Ramakrishnan [a], Ramasamy Santhosh Kumar [a], Ae Rhan Kim [a, c] and Dong Jin Yoo [a, c *]

[a] Graduate School, Department of Energy Storage/Conversion Engineering (BK21 FOUR), Jeonbuk National University, Jeonju, Jeollabuk-do 54896, Republic of Korea.

[c] Department of Life Science, R&D Education center for whole life cycle R&D of fuel cell systems, Hydrogen and Fuel Cell Research Center, Jeonbuk National University, Jeollabuk-do 54896, Republic of Korea.

Corresponding Author E-mail: djyoo@jbnu.ac.kr (Prof. Dong Jin Yoo).

Iyyappa Rajan Panneerselvam [b] and Yan Wang [b]

[b] Department of Mechanical Engineering, University of Nevada, Reno, Reno, NV, 89557, USA.

*Corresponding Email ID: djyoo@jbnu.ac.kr (Prof. Dong Jin Yoo)
Fax: +82-(0) 63-270-3909 Tel: +82-(0) 63-270- 3608

Experimental Section

Material physical characterizations

The crystal phase patterns and surface morphology of as-prepared electro composites were examined by powder X-ray diffraction (XRD, X'pert-MRD, Pro Philips), Field emission scanning electron microscopy (FE-SEM, SUPRA 40VP, ZEISS), and high-resolution transmission electron microscopy (HR-TEM, JEM-ARM 200F). Typically obtained composites topographical structures in 2D and 3D form were captured by an atomic force microscope (AFM, Bruker, multimode-8). X-ray photoelectron spectroscopy (XPS, AxisNova, Kratos, Inc.) and Raman spectroscopy (HORIBA-Lab RAM-HR equipped with 532 nm He-Ne laser) installed at Korea Basic Science Institute (KBSI) at Jeonbuk National University was used to examine the elemental composition and structural defects in the samples. The Pt loading was measured by inductively coupled plasma-optical emission spectrometry (ICP-OES, Thermo Fisher Scientific iCAP 7000 series). Thermal gravimetric analysis (TGA; TA Instruments, Q 400) was used to quantify the thermal stability at the Center for University-Wide Research Facilities at Jeonbuk National University. X-ray absorption near-edge spectroscopy (XANES), and extended X-ray absorption fine structure (EXAFS) was carried to determine the oxidation state and structural information of samples (R-XAS, Rigaku Model).

Computational details

The competition of methanol adsorption over the Pt and CoS_2 surfaces was investigated using density functional theory-based simulations as implemented in the Vienna ab initio simulation package (VASP) [1-2]. The electron-ionic core interactions were represented using the projector-augmented wave (PAW) method [2-3] and the exchange-correlation effects of electrons were obtained using Perdew, Burke, and Ernzerhof (PBE) potentials based on generalized gradient approximation (GGA) [4]. Initially, the bulk structures of Pt and CoS_2 (both ionic positions and volume) were relaxed using the conjugate gradient algorithm

method [5] with an electronic convergence of 10^{-6} eV and an ionic step convergence of 0.01 eV $Å^{-1}$. All our calculations were spin-polarized, and Gaussian smearing of 0.05 eV was used. The plane wave cut-off energy was set at 600 eV throughout the calculations. However, the Brillouin zone k-points sampling based on the Monkhorst-pack scheme was varied as follows: 13×13×13 for Pt bulk, 6×6×6 for CoS_2 bulk, and 2×2×1 for all surface/adsorption calculations.

Supplementary figures and tables

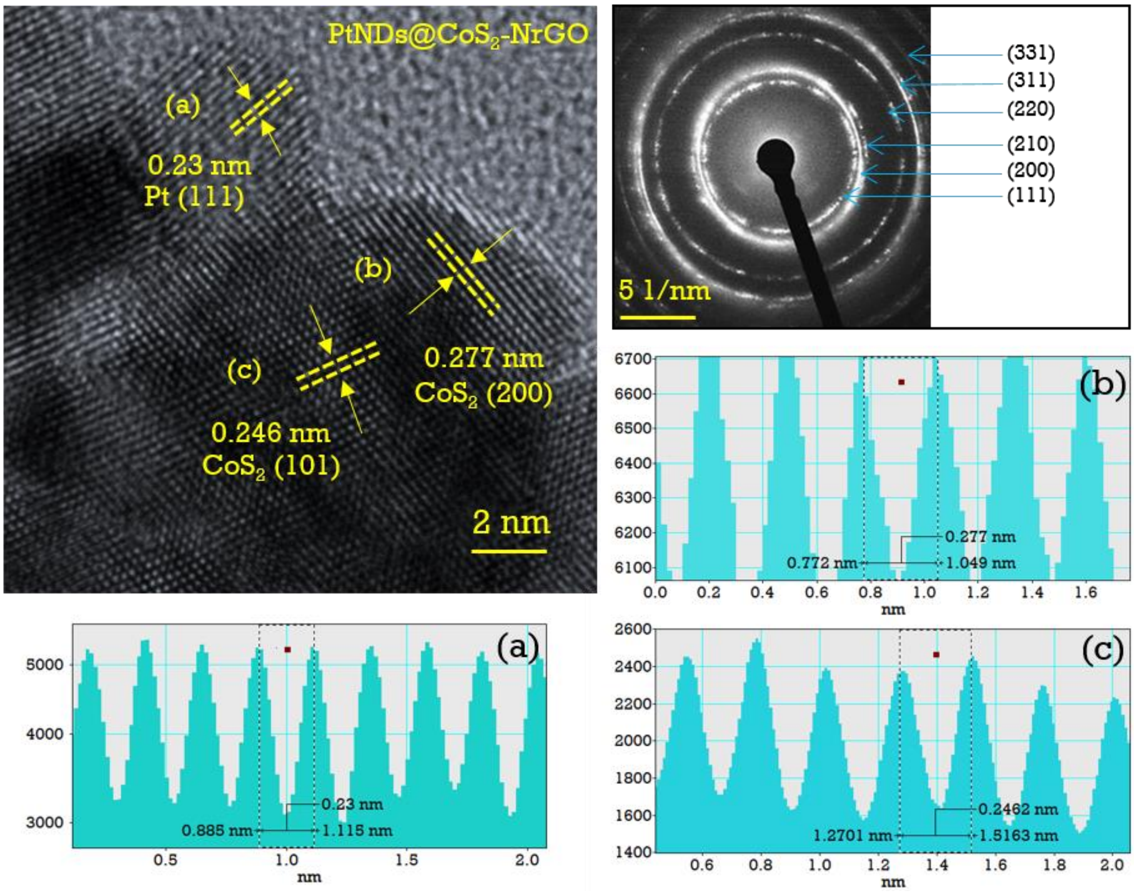

Figure S1. HR-TEM image and correspond lattice fringes evidence for the crystalline plane of PtNDs@CoS_2-NrGO composite, the corresponding areas SAED patterns. (a) Pt (111) plane, (b) CoS_2 (200) plane and (c) CoS_2 (101) plane respectively.

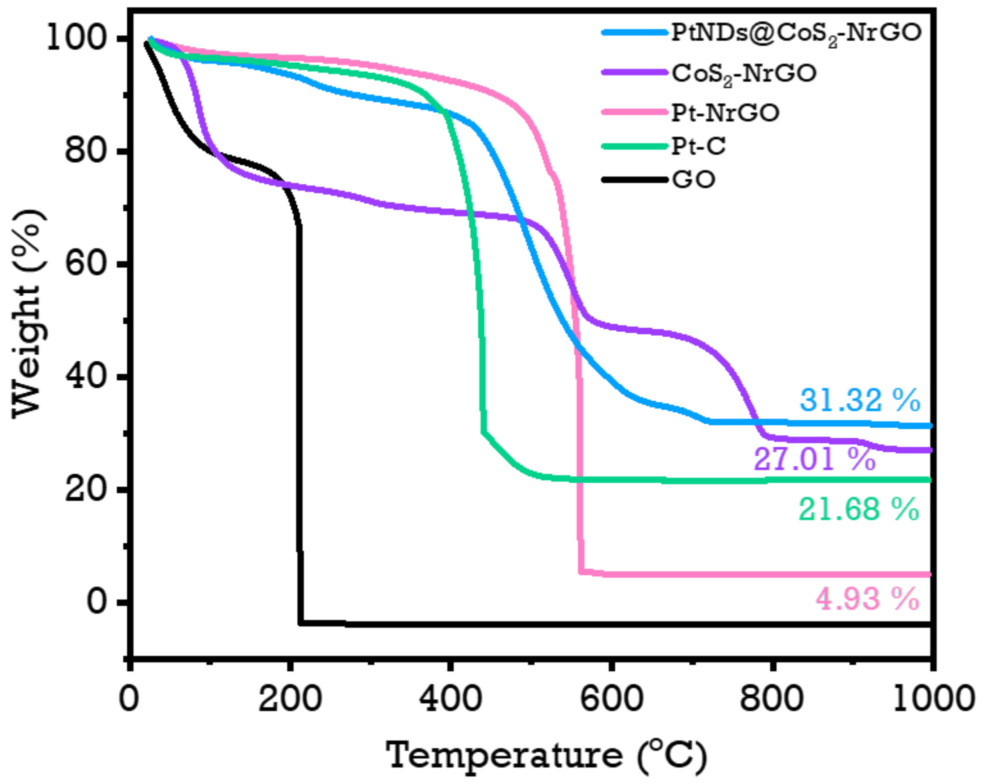

Figure S2. Thermal property analysis: Thermal gravimetry results of typically prepared PtNDs@CoS_2-NrGO, CoS_2-NrGO, Pt-NrGO, GO, and commercial Pt-C in air atmosphere conditions.

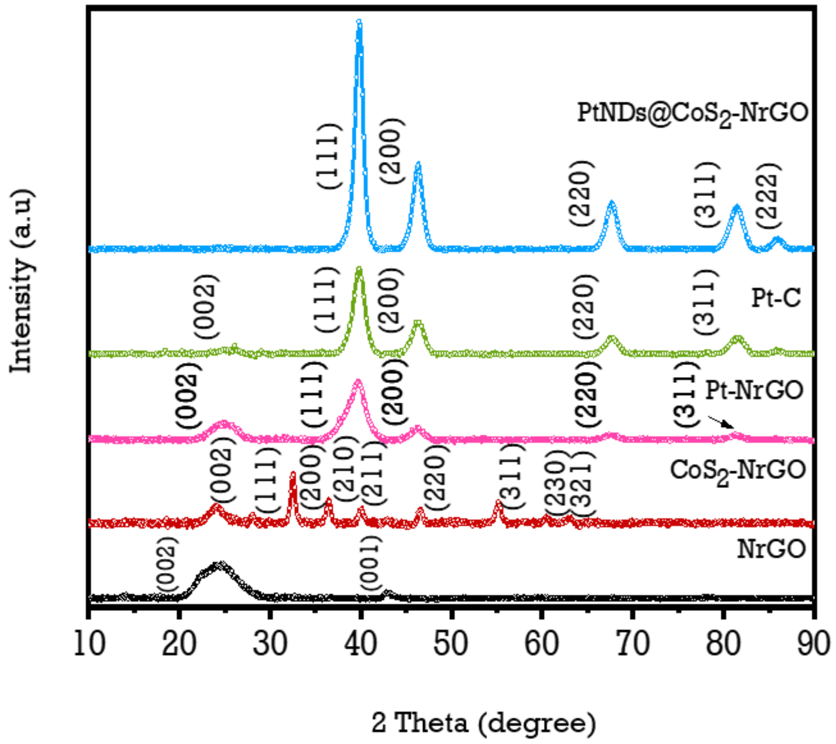

Figure S3. XRD patterns of typically prepared PtNDs@CoS_2-NrGO, commercial Pt-C, Pt-NrGO, CoS_2-NrGO, and NrGO.

Table S1. Operational conditions of above mentioned XRD analysis.

**XRD operation conditions**			
K-Alpha$_1$ wavelength	1.540598	Divergence slit	0.19 mm
K-Alpha$_2$ wavelength	1.544426	Phi	57.7
Ratio K-Alpha$_2$/K-Alpha$_1$	0.5	Time per step	85.09
Generator voltage	40	Scan range	5.006000001 – 89.50000
Tube current	30	Scan step size	0.0334225

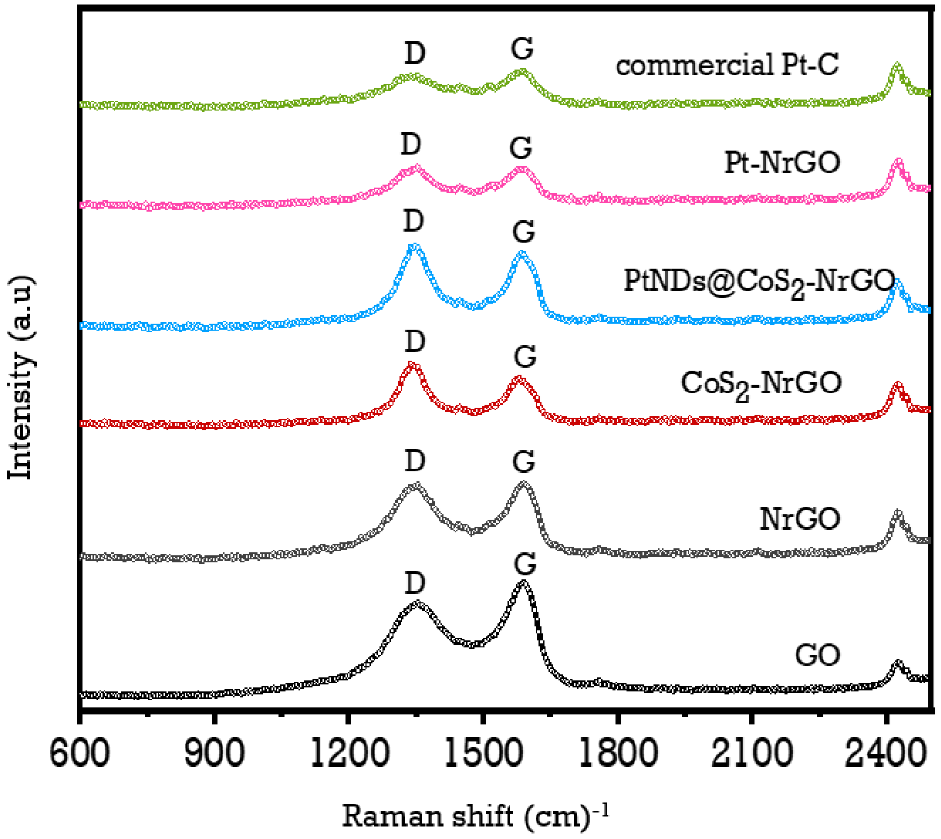

Figure S4. Raman patterns of commercial Pt-C, Pt-NrGO, PtNDs@CoS_2-NrGO, CoS_2-NrGO, NrGO, and GO, respectively.

Table S2. Calculated I_D/I_G ratio values of commercial Pt-C, Pt-NrGO, PtNDs@CoS_2-NrGO, CoS_2-NrGO NrGO, and GO from Raman spectrum analysis.

S. No	Composites	I_D/I_G Ratio
1	Commercial Pt-C	0.99
2	Pt-NrGO	1.00
3	PtNDs@CoS_2-NrGO	1.04
4	CoS_2-NrGO	1.02
5	NrGO	0.99
6	GO	0.83

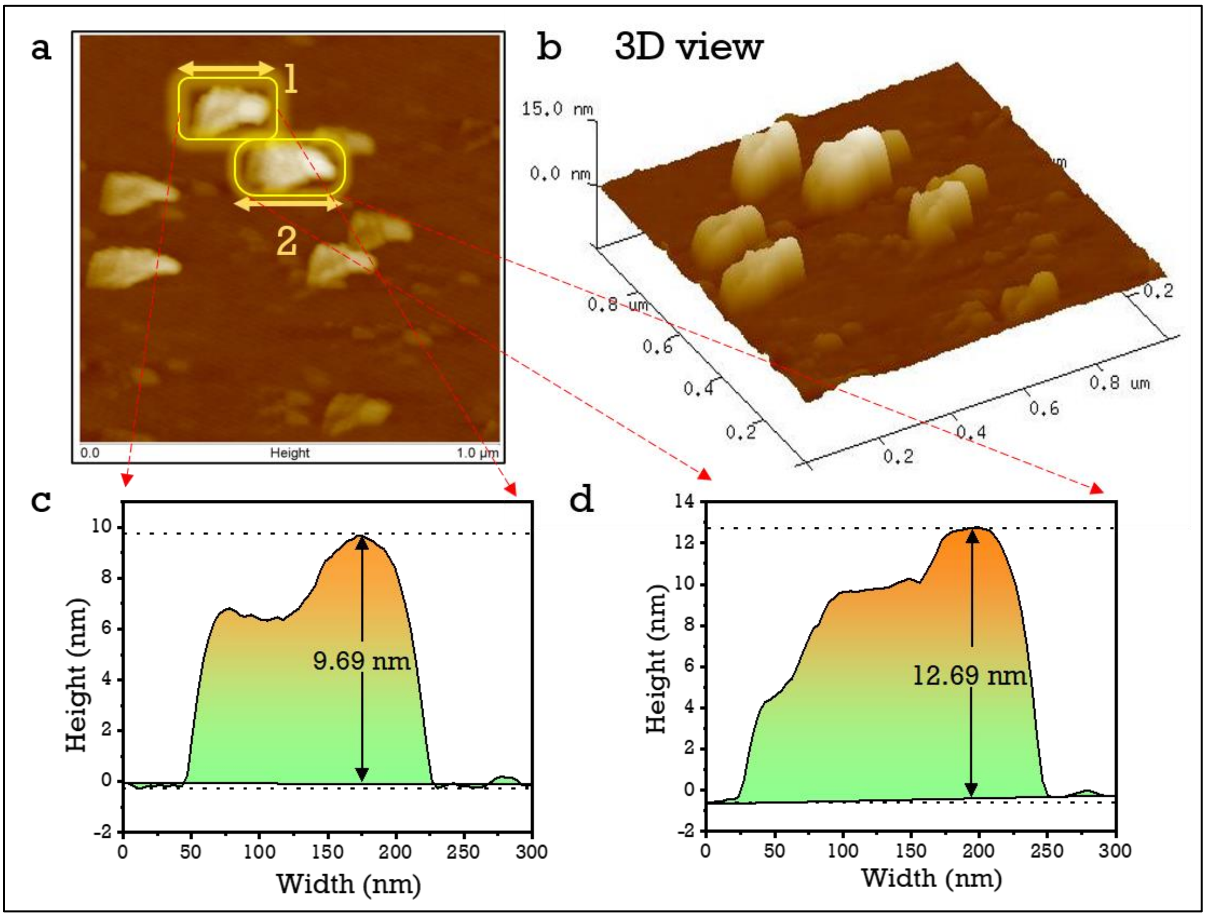

Figure S5. Atomic force microscopy images to examine the topography of PtNDs@CoS_2-NrGO. (a) 2D, (b) 3D views projections of PtNDs@CoS_2-NrGO, and (c and d) height profiles of PtNDs@CoS_2-NrGO. Which is used to analyze the width of the material. The 2D and 3D images in (a and b) exhibit the roughness of the material. Which evidence the nano Pt particle dispersion on both CoS_2 nanoparticles and N-doped graphene surfaces, the average width of the PtNDs@CoS_2-NrGO material is measured to be 200-250 nm in Figure S5(c and d). This topographical analysis further supports the successful material interactions of Pt and CoS_2-NrGO.

Table S3. Bulk composition weight percentage of PtNDs@CoS_2-NrGO and CoS_2-NrGO

Composition weight (%)						
**Composites**	**EDS**				**ICP-OES**	
Elements	Pt	Co	S	C	Pt	Co
PtNDs@CoS_2-NrGO	14.2	9.79	5.68	57.3	15.9	5.7
CoS_2-NrGO	-	27.86	36.8	34.98	-	32.5

electrocatalysts were evaluated from STEM-EDS and ICP-OES results.

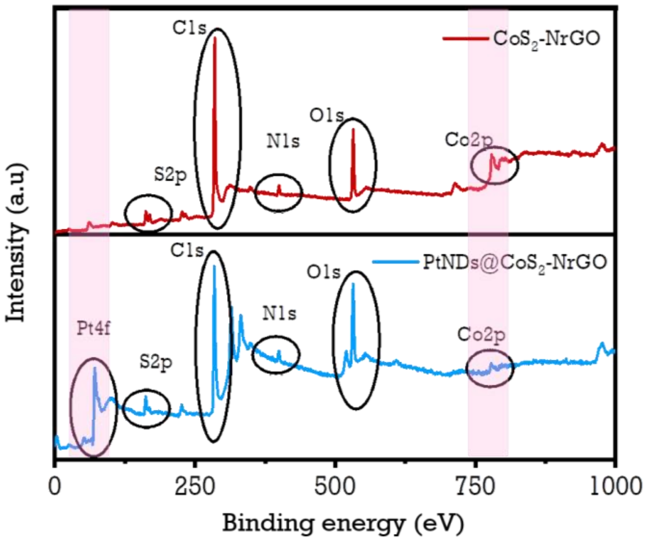

Figure S6. High resolution XPS survey spectrum of CoS_2-NrGO and PtNDs@CoS_2-NrGO electrocatalyst.

Name	Peak BE (eV)	FWHM (eV)	Area (P) CPS.eV	Atomic (%)
S 2p	168.92	2.28	41254.57	4.69
C 1s	284.48	1.11	321674.8	69.88
N 1s	401.39	2.58	15277.91	2.26
O 1s	532	2.66	196863.2	10.71
Co 2p	781.72	5.23	23964.28	12.45

Table S4. Elemental composition of CoS_2-NrGO evaluated by XPS.

Name	Peak BE (eV)	FWHM (eV)	Area (P) CPS.eV	Atomic (%)

Table S5. Elemental composition of PtNDs@CoS_2-NrGO evaluated by XPS.

Pt 4f	70.91	1.99	295558.2	7.22
S 2p	161.63	2.75	32812.23	5.41
C 1s	283.85	1.48	200732.7	65.1
N 1s	399.12	2.59	18137.86	3.91
O 1s	531.49	3.13	126714	11.53
Co 2p	777.71	1.34	30171.86	6.82

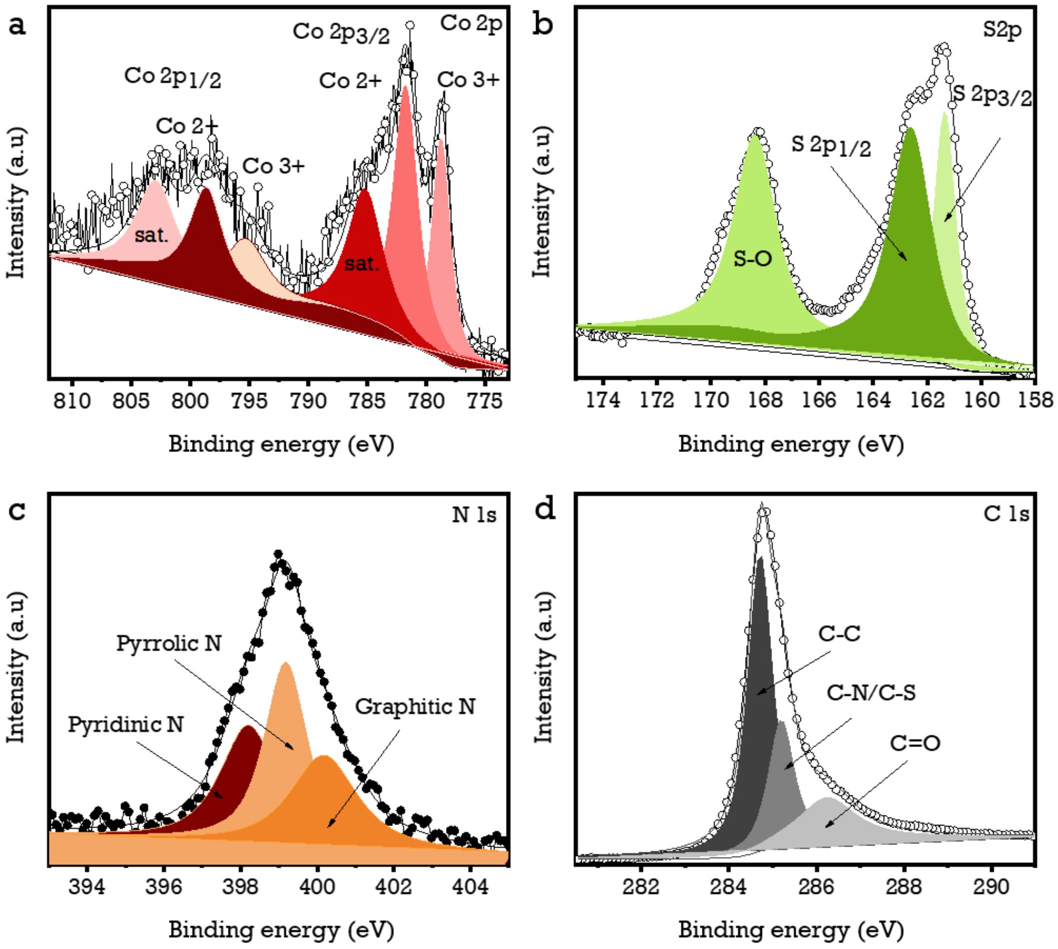

Figure S7. High-resolution XPS deconvolution spectra of CoS_2-NrGO composite elements (a) Co 2p, (b) S 2p, (c) N 1s and (d) C 1s respectively.

Deeper elemental composition discussions of Co 2p and S 2p elements of CoS_2-NrGO composite were compared with Co 2p and S 2p elements of PtNDs@CoS_2-NrGO in the result and discussions part. Further the high-resolution deconvolutions peaks of N 1s in Figure S7c shows the three major peaks at 398.4 eV, 399.3 eV, and 400.5 eV attributed to pyridinic-N pyrrolic-N and graphitic-N, respectively. Fig S7d shows the high-resolution deconvolutions

peaks of C1s where three major C-C, C-N/C-S, C=O peak positions belong to the binding energies of 284.6 eV, 285.9 eV, and 286.2 eV, respectively.

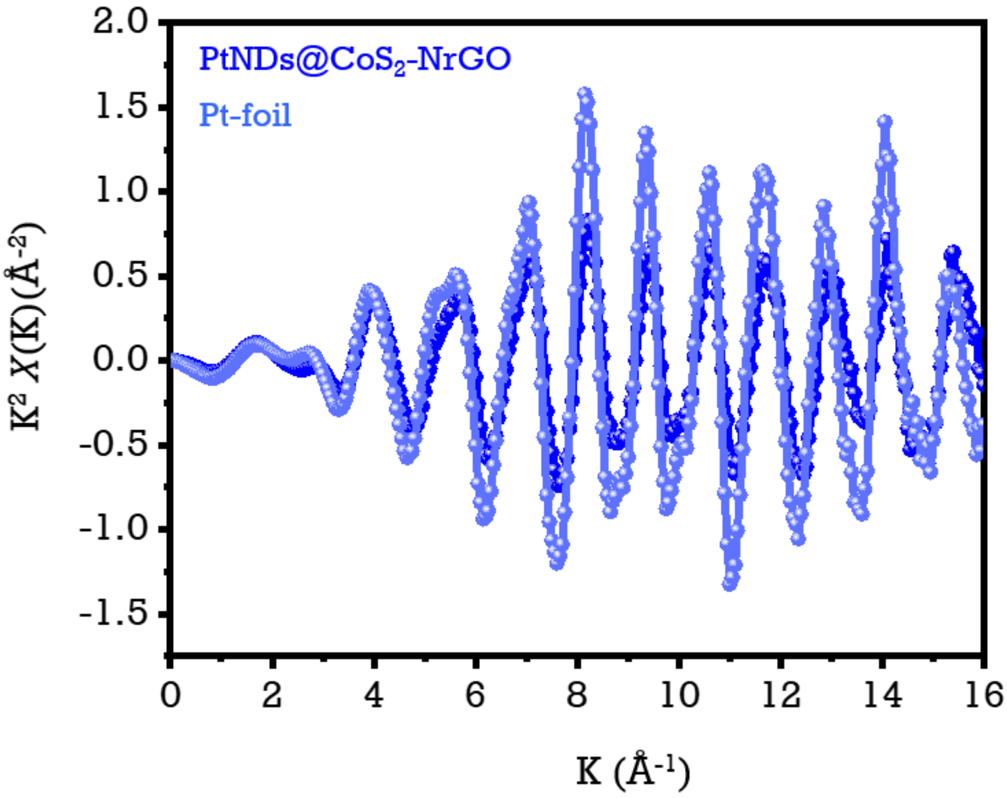

Figure S8. Pt L3-edge EXAFS oscillations in k space (k^2-weighting) of PtNDs@CoS_2-NrGO and Pt-foil.

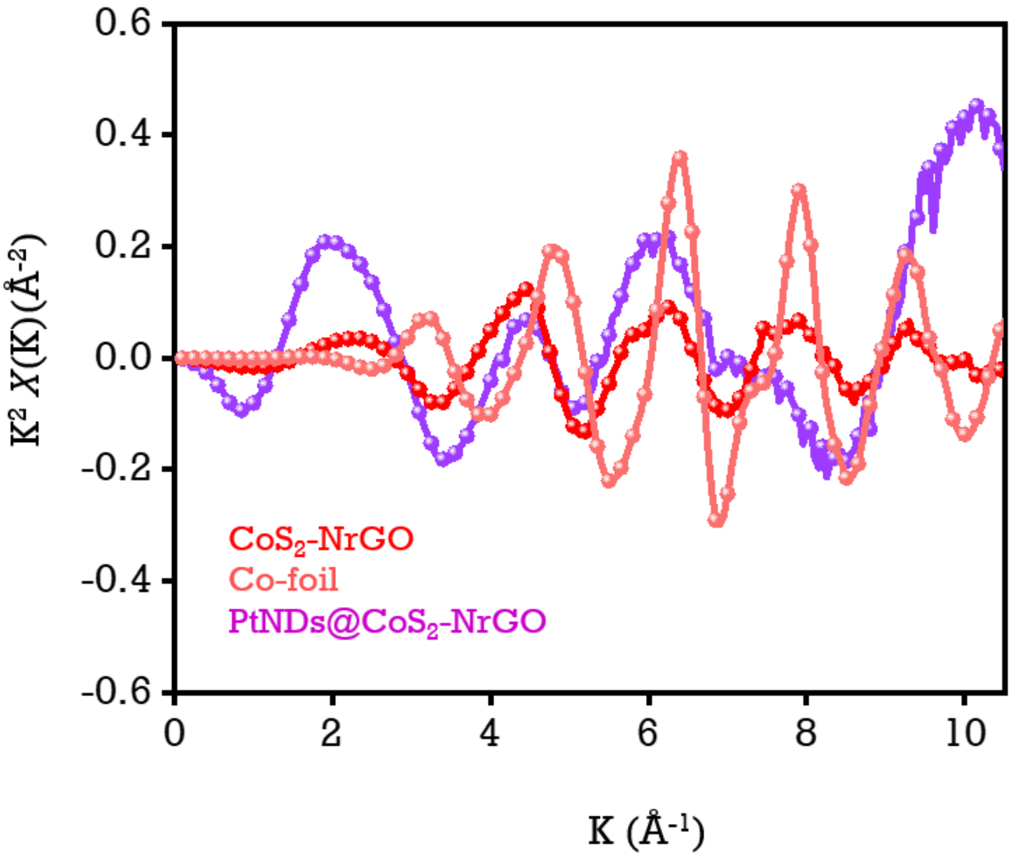

Figure S9. Co K-edge EXAFS oscillations in k space (k^2-weighting) of CoS_2-NrGO, PtNDs@CoS_2-NrGO, and Co-foil.

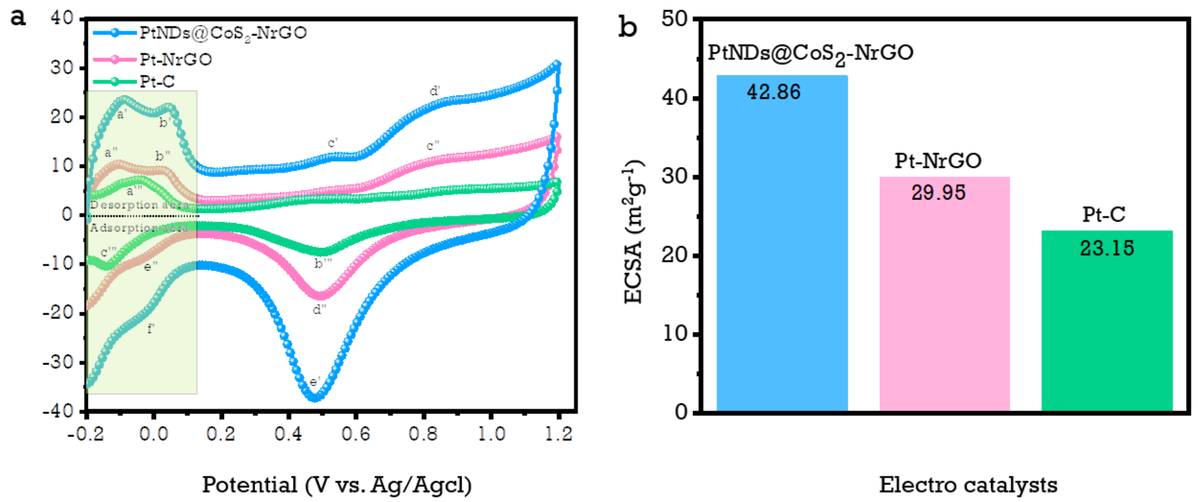

Figure S10. Respected cyclic voltammogram responses of PtNDs@CoS_2-NrGO, Pt-NrGO, and commercial Pt-C in 0.5 M H_2SO_4 analyte at a sweep rate of 50 mV s^{-1} (a). Corresponding calculated ECSA value bar charts (b).

To analyze the background behaving property of PtNDs@CoS_2-NrGO, Pt-NrGO, and commercial Pt-C composites, hydrogen adsorption and desorption calculation were evaluated in Figure S10 using the ECSA calculations in a sweeping range of -0.2 to 1.2 V vs. Ag/AgCl at a sweep rate of 50 mV s^{-1} in 0.5 M H_2SO_4 analyte solutions. In which six major peaks were observed on the PtNDs@CoS_2-NrGO electrocatalyst, at -0.0937 V, 0.0508 V, 0.5359 V, 0.8377 V, 0.4795 V, and -0.02156 V which respect to six major regions of electrochemical redox process denoted as (a'- f') in Figure S10(a). These are attributed to the hydrogen desorption on the PtNDs surfaces in (a' to b') and create a sharp reduction in e' further the better hydrogen adsorption on f'. Compared to the Pt-NrGO and commercial Pt-C hydrogen adsorption and desorption in the regions of (a" to e") and (a"' to c"'). Associated electrochemical active surface area (ECSA) calculations were evaluated by conventional calculations method[6], and the resulted values are sequenced to PtNDs@CoS_2-NrGO > Pt-NrGO > Pt-C in Figure S10(b). These resulting values strongly suggest that PtNDs@CoS_2-NrGO has highly active surface areas owing to their successful formation of Pt nanodendrites on CoS_2 surfaces.

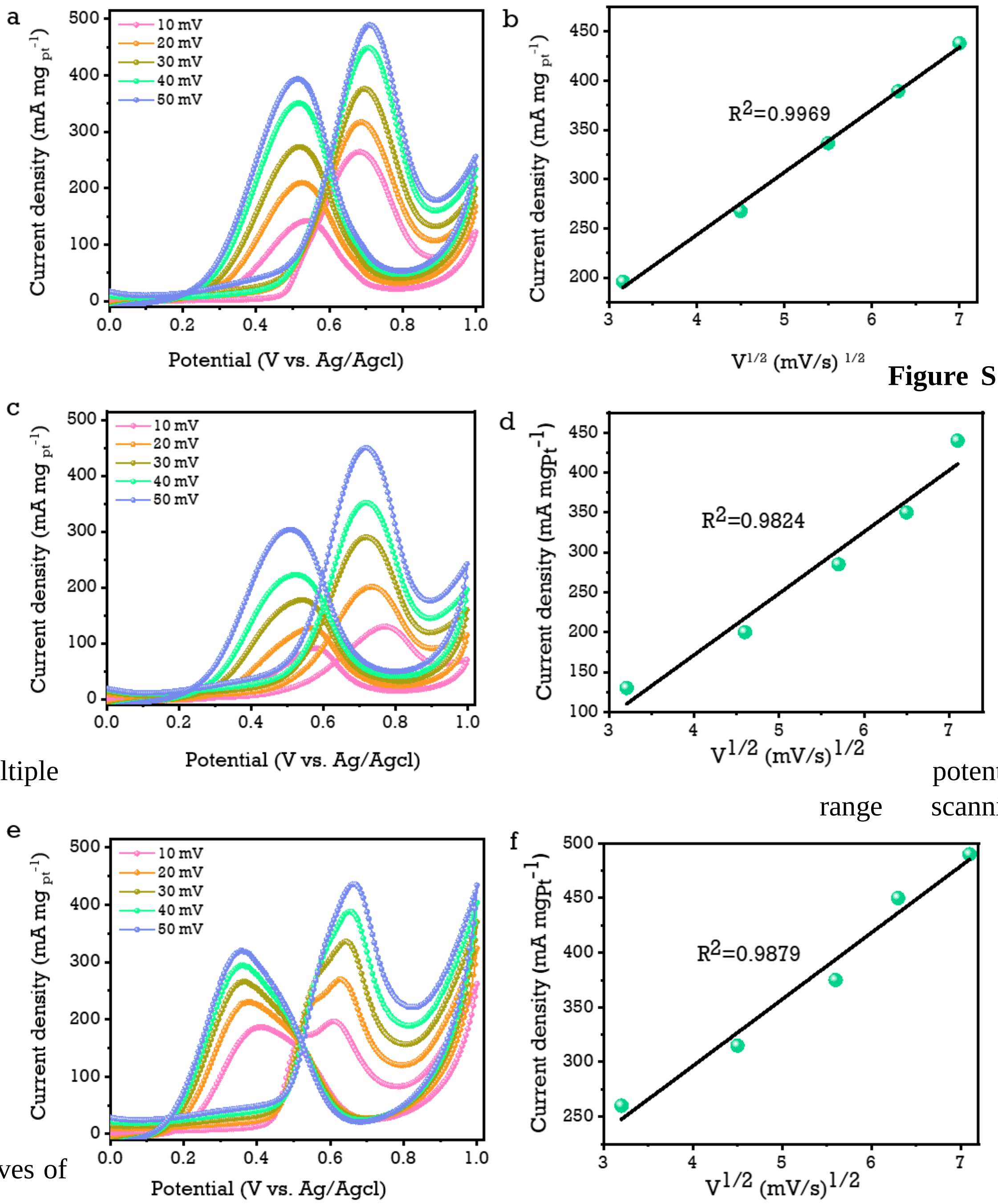

Figure S11. Multiple potential range scanning curves of PtNDs@CoS_2-NrGO in (a) methanol, (c) ethylene glycol, (e) glycerol electrolytes and their corresponding calculated R^2 values in (b, d, f), respectively.

Further, the diffusion-controlled current behavior was examined through multiple CV cycles analysis. Figure S11(a, c, e) shows the multiple potential CV responses of methanol, ethylene glycol, and glycerol in 0.5 M H_2SO_4 solution. Corresponding R^2 calculations in Figure S11(b, d, f) shows the values of 0.9969, 0.9824, and 0.9879, respectively.

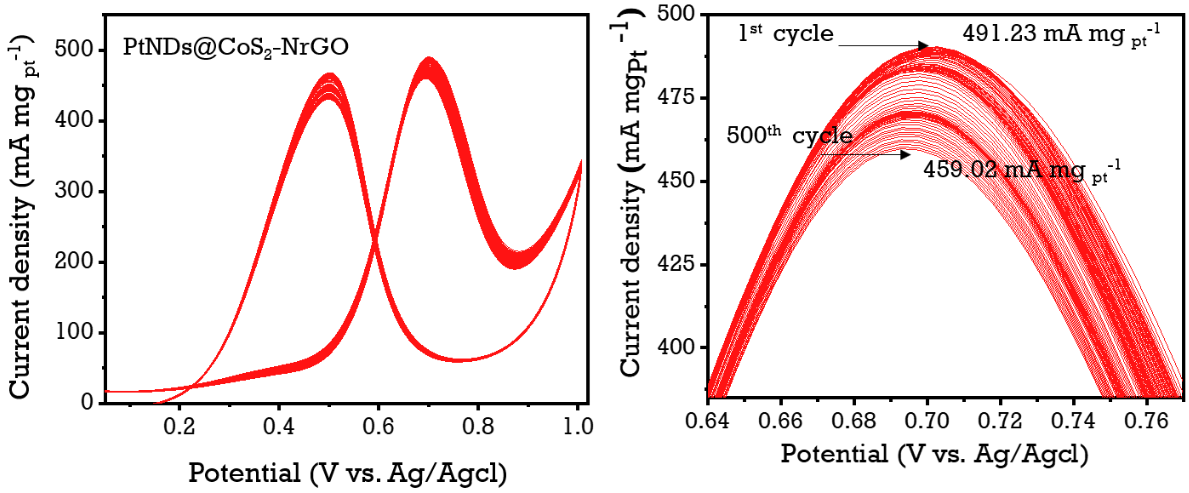

Figure S12. Long-term cyclic voltammetry test for PtNDs@CoS_2-NrGO electrocatalyst at a scan rate of 50 mV in 1 M methanol in 0.5 M H_2SO_4 electrolyte solution. This shows the impairing of J_f/J_b intensity ratio by strongly adsorbed CO intermediates, and this keeps the CO tolerance from 1.13 to 0.918 after the 500th sweep.

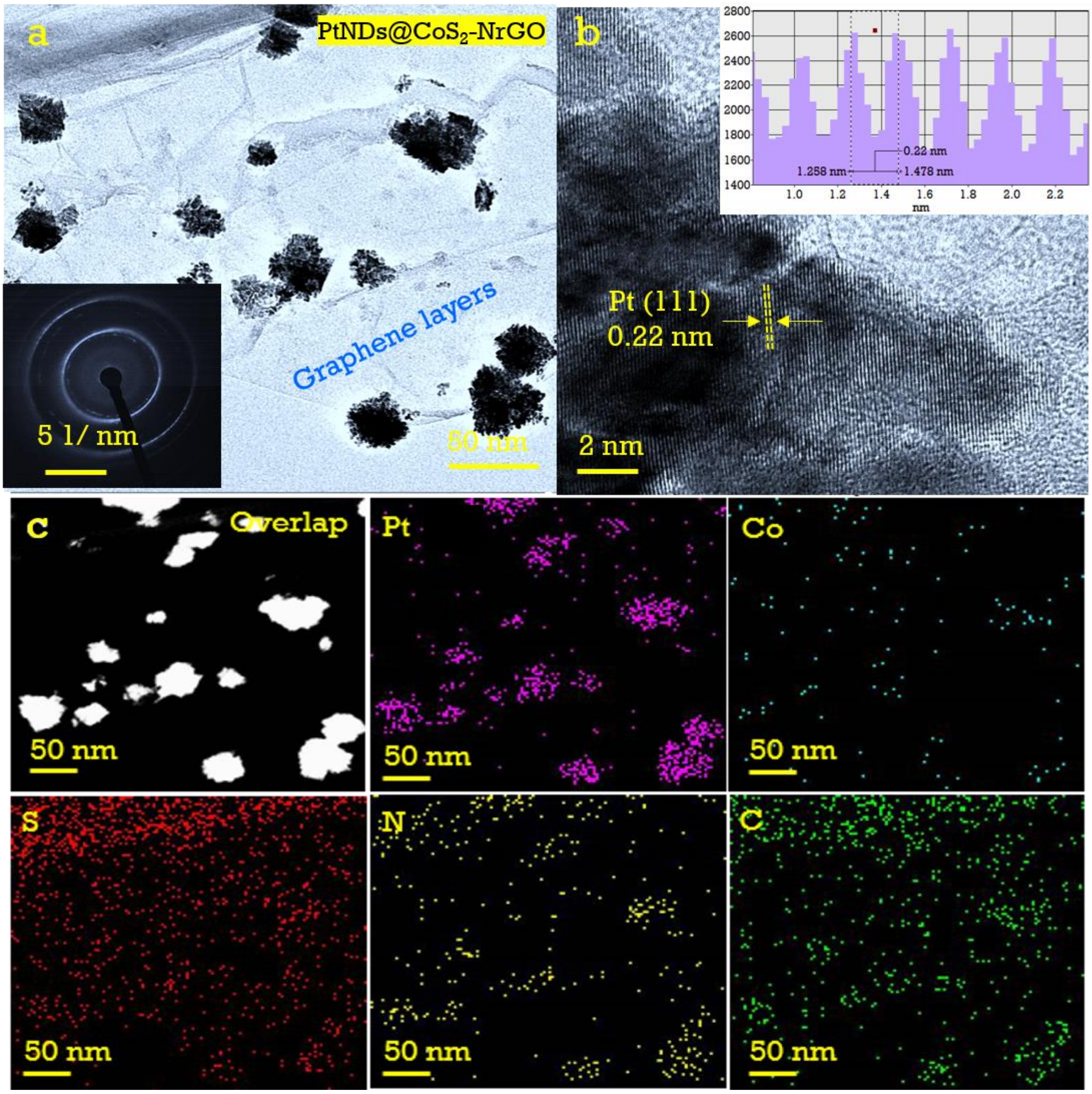

Figure S13. Post morphology analysis of PtNDs@CoS_2-NrGO electrocatalysts after 500 CV cycles in 1 M MeOH + 0.5 M H_2SO_4 electrolyte solution. (a) CS-TEM (inset: corresponding SAED) (b) HR-TEM images of PtNDs@CoS_2-NrGO. (c) HAADF-STEM elemental mapping of PtNDs@CoS_2-NrGO concerning Pt-k, Co-k, S-k, N-k, C-k.

The post morphology analysis of PtNDs@CoS_2-NrGO was investigated by using TEM analysis. The sample was collected from the GCE electrode after 500 cycles of CV. Figure S13a shows that there are no significant morphology changes in Pt nanodendrites at $CoS_{2,}$ which is clearly attached to the NrGO sheet. This confirms the morphology stability of PtNDs@CoS_2-NrGO after 500 CV cycles. Furthermore, we confirmed the crystallinity of PtNDs@CoS_2-NrGO using SAED and HRTEM analysis. The SAED result shows a bright

ring with a dotted spot (insert image of Fig S13a), confirming the crystallinity of the catalysts. HR-TEM shows the lattice fringes of PtNDs are about 0.22 nm, corresponding to the planes of (1 1) as shown in Fig 13b. The long-term cycling test, Figure S13c shows the HAADF-STEM elemental mapping of PtNDs@CoS_2-NrGO, the presence of elements Pt, Co, S, N, and C. There are only changes in Co composition as compared to before the long-term cycling durability test.

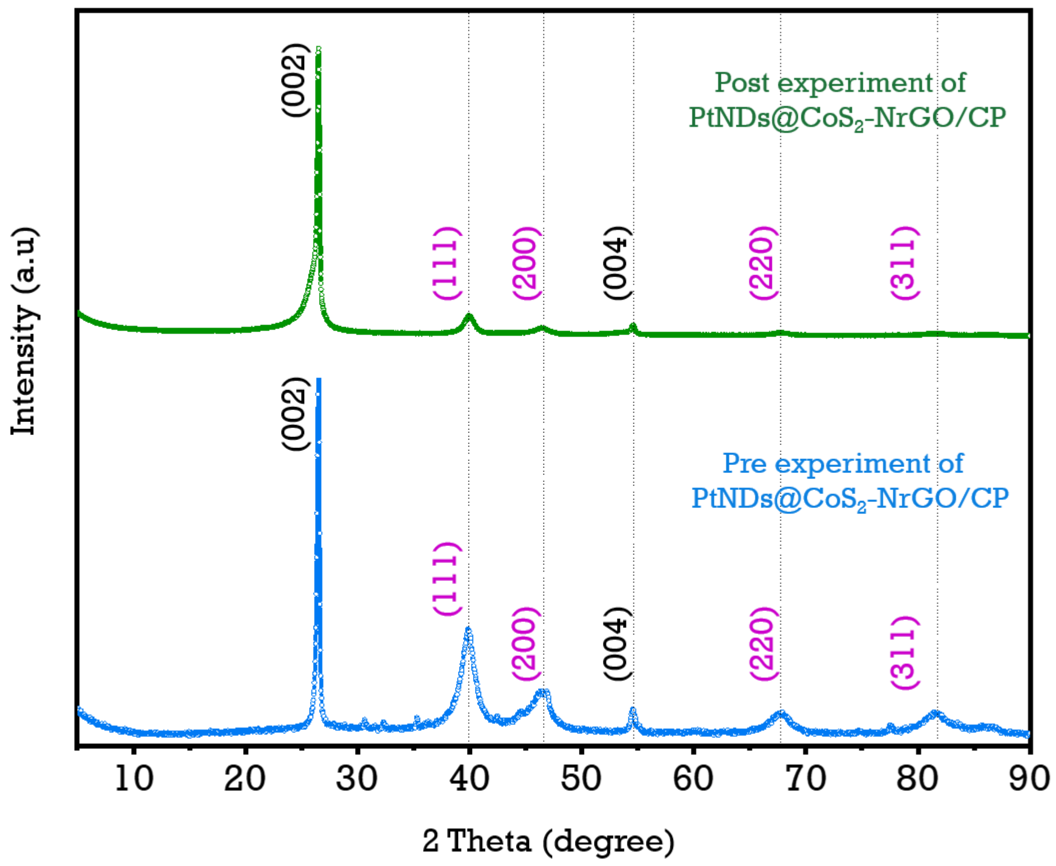

Figure S14. Post morphology XRD analysis of PtNDs@CoS_2-NrGO electrocatalysts coated on carbon paper (CP) after long-term chronoamperometry in 1 M MeOH + 0.5 M H_2SO_4 electrolyte solution.

The post-experiment crystallinity nature of PtNDs@CoS_2-NrGO was further investigated after long-term chronoamperometry tests using XRD analysis in Figure S14. Long-term chronoamperometry was performed in a 1 M MeOH + 0.5 M H_2SO_4 electrolyte solution with the appropriate amount of PtNDs@CoS_2-NrGO coated on the carbon paper (CP). The XRD pattern of PtNDs@CoS_2-NrGO/CP (before long-term stability) displays major Pt characteristic peaks at 39.9º, 46.6º, 67.7º, and 81.4º, correspond to (111), (200), (220), and (311) planes respectively, which is well-matched with standard cubic Pt (JCPDS no. 65-2868). After the long-term stability test, the XRD pattern of the PtNDs@CoS_2-NrGO/CP confirms that only the peak intensity was reduced without any changes in the peak position, as shown in figure S14. Therefore, the PtNDs@CoS_2-NrGO electrocatalyst exhibits good stability towards methanol electrooxidation.

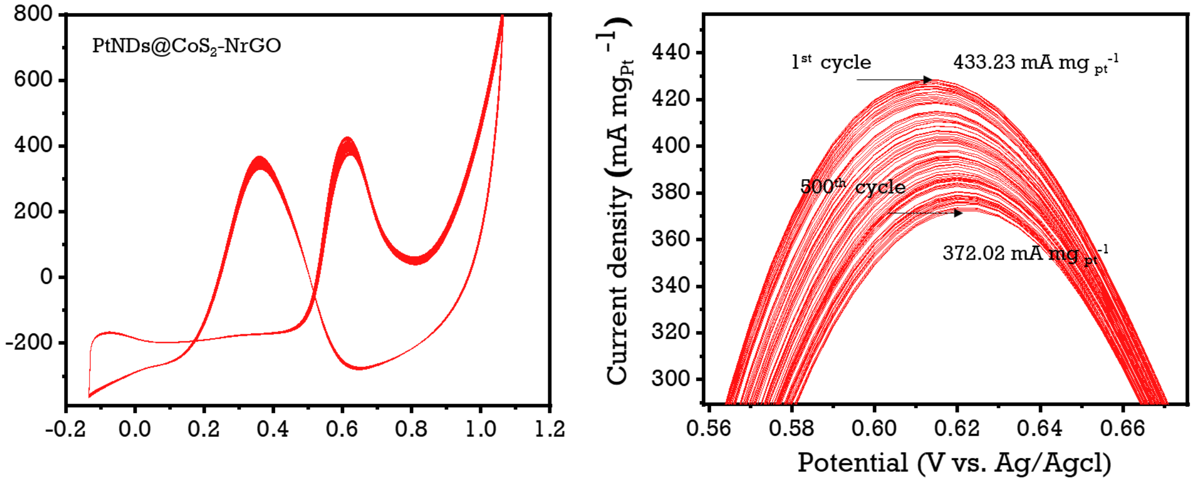

Figure S15. Long-term cyclic voltammetry test for PtNDs@CoS_2-NrGO at a scan rate of 50 mV in 1 M ethylene glycol in 0.5 M H_2SO_4 solution. This shows the impairing of J_f/J_b intensity ratio by strongly adsorbed CO intermediates, and this keeps the CO tolerance from 1.3 to 1.00 after the 500th sweep.

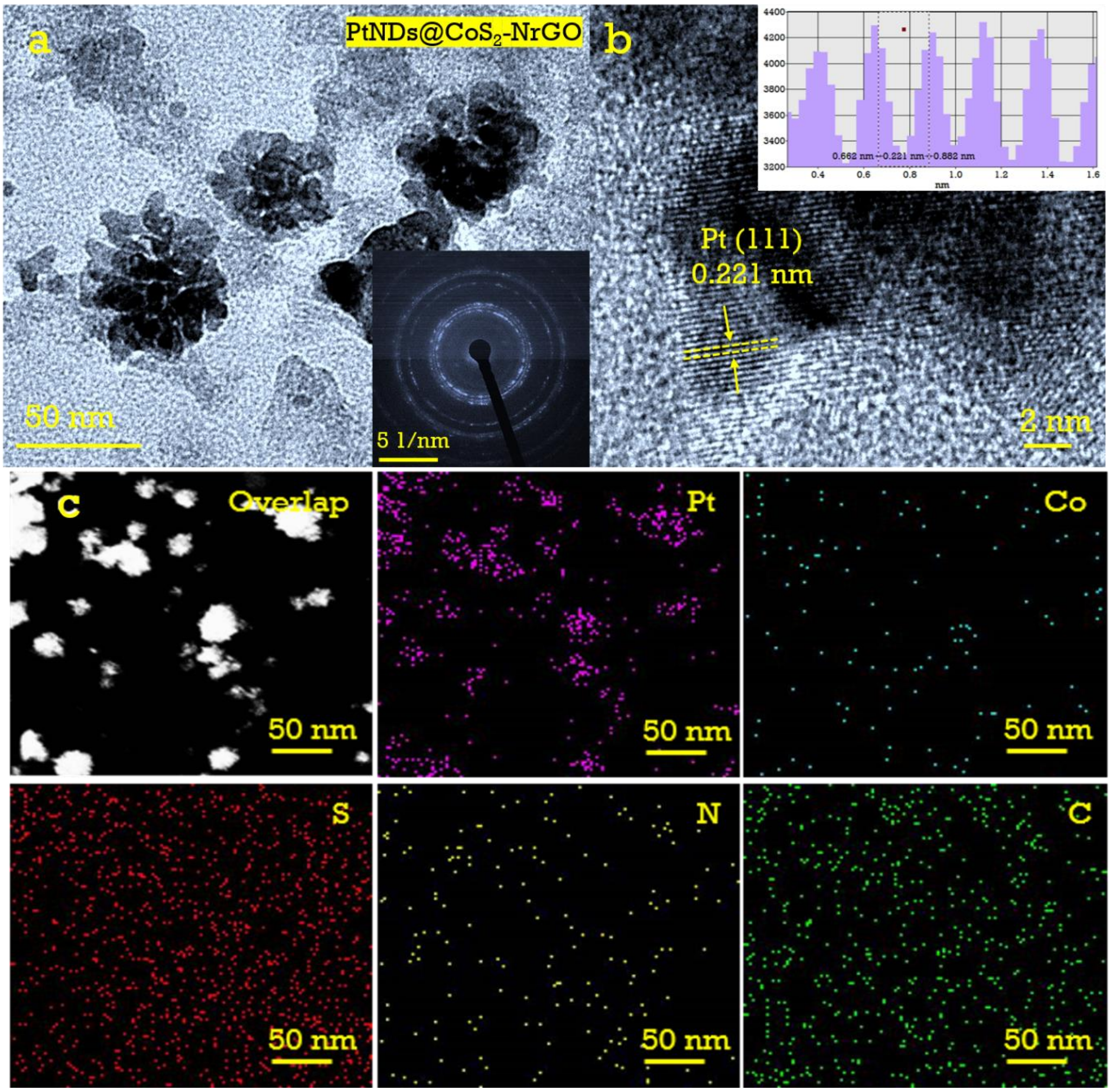

Figure S16. Post morphology analysis of PtNDs@CoS_2-NrGO after 500 CV cycles in 1 M ethylene glycol + 0.5 M H_2SO_4 electrolyte solution. (a) CS-TEM (b) HR-TEM (inset: corresponding FFT) images of PtNDs@CoS_2-NrGO. (c) HAADF-STEM elemental analysis of PtNDs@CoS_2-NrGO concerning Pt-k, Co-k, S-k, N-k, C-k.

Figure S16 shows the morphology analysis of PtNDs@CoS_2-NrGO after a long-term cycling durability test with 1 M ethylene glycol in an acidic medium. The TEM image of Figure S16 reveals that there is a slight change in the morphology of PtNDs@CoS_2 on the NrGO sheet, while the SAED pattern (insert image of Figure S16a) of the PtNDs@CoS_2-NrGO shows good crystalline nature. The HR-TEM image shows the clear lattice fringes of PtNDs are

about 0.22 nm (planes of (111) and as shown in Figure S16b, there are no changes in the crystallinity of the electrocatalyst during the long-term cycling durability test. After the long-term cycling test, Figure S16c shows the HAADF-STEM elemental mapping of PtNDs@CoS_2-NrGO and the presence of elements Pt, Co, S, N, and C. There are only changes in Co and N composition as compared to before the long-term cycling durability test.

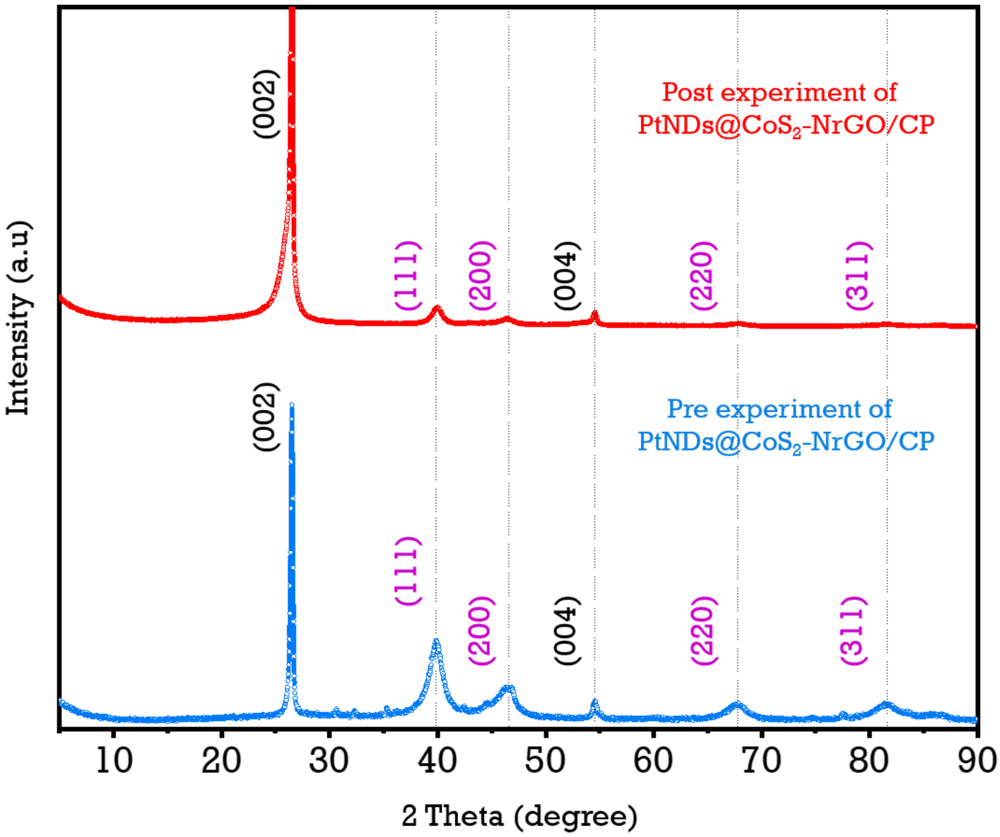

Figure S17. Post morphology XRD analysis of PtNDs@CoS_2-NrGO electrocatalysts coated on carbon paper (CP) after long term chronoamperometry in 1 M ethylene glycol + 0.5 M H_2SO_4 electrolyte solution.

The post-experimental crystallinity nature of PtNDs@CoS2-NrGO was further investigated by using XRD analysis after a long-term chronoamperometry test in a 1 M ethylene glycol + 0.5 M H_2SO_4 electrolyte solution and an appropriate amount of PtNDs@CoS_2-NrGO electrocatalysts coated on the carbon paper (CP) (see Figure S17). The XRD pattern of PtNDs@CoS_2-NrGO/CP (before long-term stability) displays major Pt characteristic peaks at 39.8°, 46.6°, 67.5°, and 81.4° correspond to (111), (200), (220), (311), and (222) planes respectively, which is fine matched with standard cubic Pt (JCPDS no. 65-2868). The additional peaks at 26.4 and 54.5° belong to (002) and (004) planes of graphitic carbon from the carbon sheet. After the long-term stability test, the XRD pattern of PtNDs@CoS_2-NrGO/CP confirms that only the peak intensity was reduced without any changes in the peak

position, as shown in Figure S17. Which demonstrates the good electrocatalyst stability during electrooxidation of ethylene glycol.

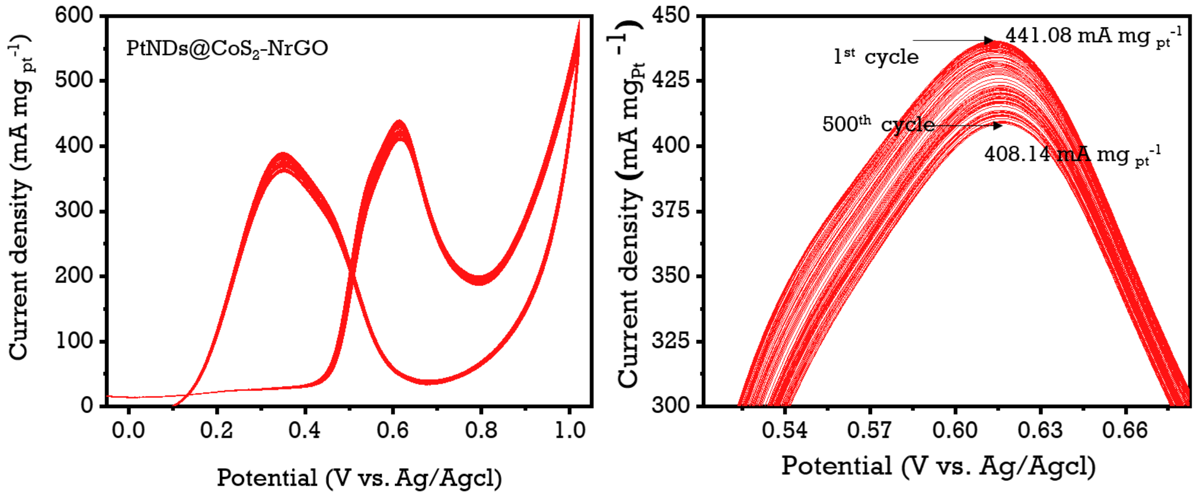

Figure S18. Long-term cyclic voltammetry test for PtNDs@CoS_2-NrGO electrocatalyst at a scan rate of 50 mV in 1 M glycerol in 0.5 M H_2SO_4 solution. This shows the impairing of J_f/J_b intensity ratio by strongly adsorbed CO intermediates, and this keeps the CO tolerance from 1.22 to 1.04 after the 500th sweep.

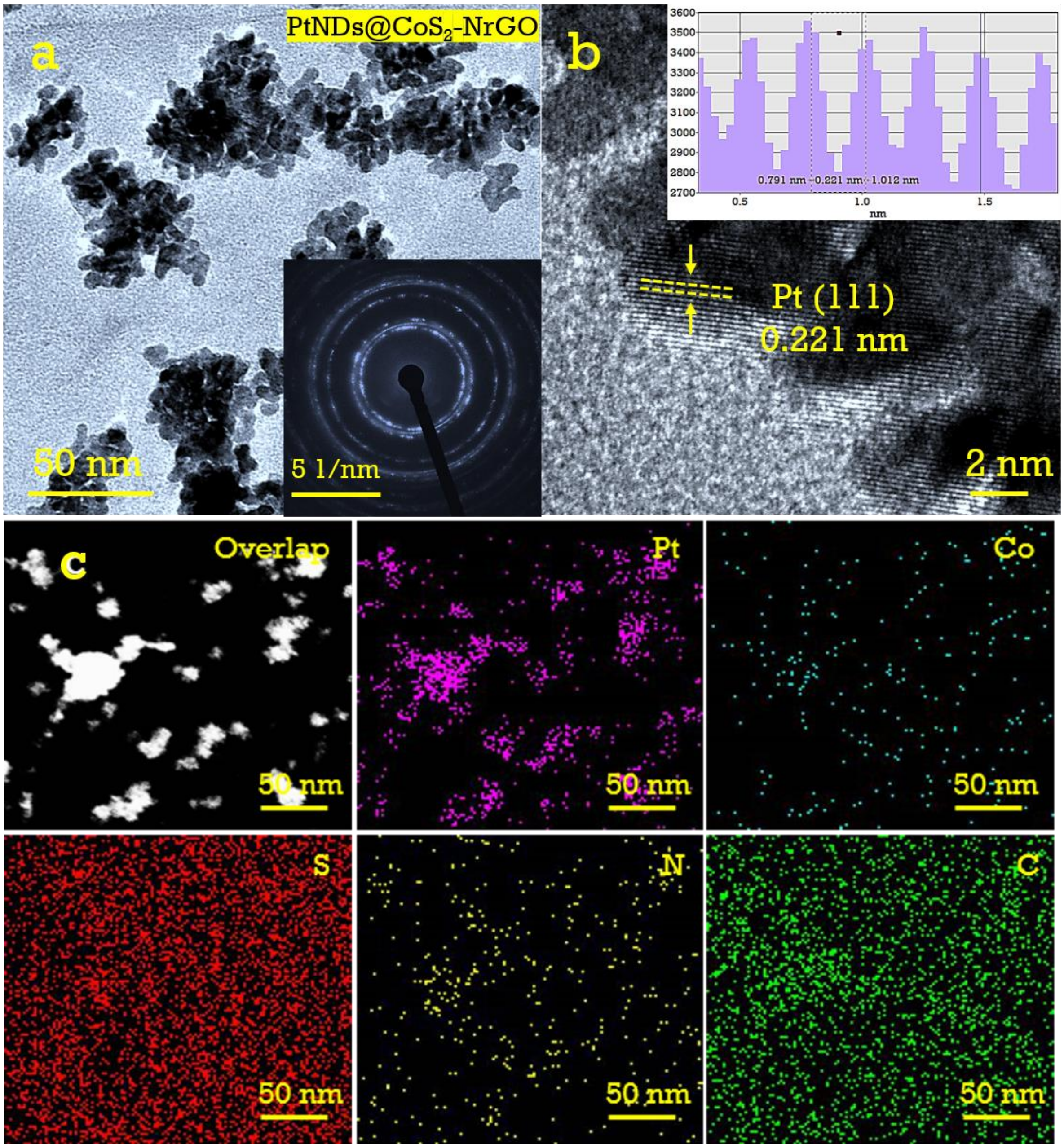

Figure S19. Post morphology analysis of PtNDs@CoS_2-NrGO after 500 CV cycles in 1 M glycerol + 0.5 M H_2SO_4 electrolyte solution. (a) CS-TEM (b) HR-TEM (inset: corresponding FFT pattern) images of PtNDs@CoS_2-NrGO. (c) HAADF-STEM elemental analysis of Pt@CoS_2-NrGO concerning Pt-k, Co-k, S-k, N-k, C-k.

Figure S19 shows the post morphology analysis of PtNDs@CoS_2-NrGO electrocatalyst in the glycerol oxidation reaction after 500 long-term cyclic stability tests. In Figure S19(a), TEM images of PtNDs are slightly agglomerated and insert images of SAED show the good crystallinity after 500 cycles of CV. Figure S19(b) reveals the existence of Pt (111) with lattice fringe distance about 0.22 nm after 500 cycles of CV, demonstrating there are no

changes of Pt (111) planes and the good stability of Pt active sites in catalysts. The HAADF-STEM elemental mapping of PtNDs@CoS_2-NrGO, which demonstrates the presence of elements Pt, Co, S, N, and C, is shown in Figure S19c. Only the composition of Co has changed since the long-term cycling durability test.

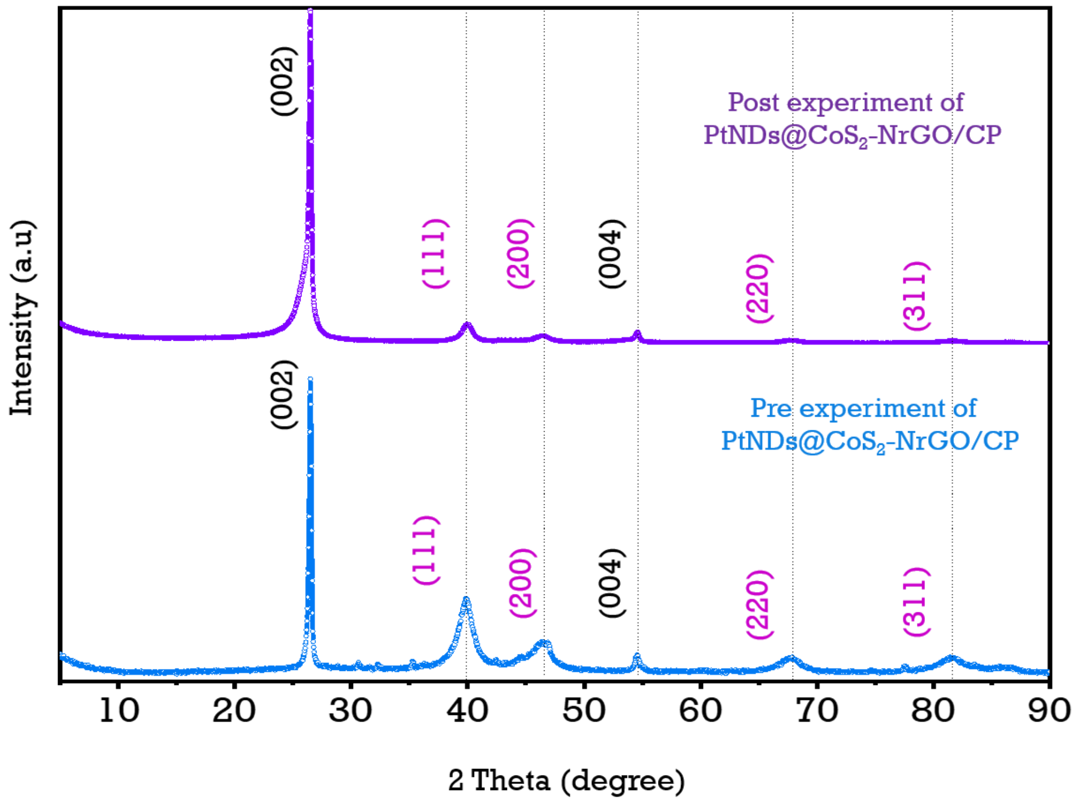

Figure S20. Post morphology XRD analysis of PtNDs@CoS_2-NrGO electrocatalysts coated on carbon paper (CP) after long-term chronoamperometry in 1 M glycerol + 0.5 M H_2SO_4 electrolyte solution.

The post-experimental crystallinity nature of PtNDs@CoS2-NrGO was further investigated by using XRD analysis after a long-term chronoamperometry test in a 1 M glycerol + 0.5 M H_2SO_4 electrolyte solution and an appropriate amount of electrocatalyst coated on the carbon paper (CP) (see Figure S20). The XRD pattern of PtNDs@CoS_2-NrGO/CP (before long term stability) displays major Pt characteristic peaks at 39.8°, 46.6°, 67.5°, and 81.4° correspond to (111), (200), (220), and (311), planes respectively, which is fine matched with standard cubic Pt (JCPDS no. 65-2868). The additional peaks at 26.4 and 54.5° belong to (002) and (004) planes graphitic carbon from carbon sheet substrate. After the long-term stability test, the XRD pattern of the PtNDs@CoS_2-NrGO/CP confirms that only the peaks intensity were reduced without any change in peaks position, as shown in Figure S20. These results are indicating that excellent electrocatalyst stability during electrooxidation of glycerol.

Table S6. Comparison table of methanol (CH_3OH) oxidation reaction (MOR) performances on PtNDs@CoS_2-NrGO electrocatalyst and recently reported nanocomposites in both alkaline and acid media.

**Electrocatalyst**	**Fuel**	**Electrolyte**	**ECSA**	**Scan rate (mV s^{-1})**	**Onset potential**	**Peak current density**	**references**
$PdRh_3$/C	0.5 M CH_3OH	0.5 M KOH	-	20	0.5 V	369.2 mA $mg^{-1}{}_{Pd}$	[7]
NiSn-1:2	0.5 M CH_3OH	0.5 M KOH	-	50	0.51 V	416.8	[8]
$Pt_{2.0}Au$/RGO	2.0 M CH_3OH	0.5 M KOH	11.14 (m^2 $g^{-1}{}_{Pt}$)	50	0.48 V	410 (m^2 $g^{-1}{}_{Pt}$)	[9]
PdRh+20 mg KI	1.0 M CH_3OH	1.0 M NaOH	42.01 (m^2 $g_{Pd}{}^{-1}$)	100	0.40 V	329.87 (m^2 $g_{Pd}{}^{-1}$)	[10]
$Pd_{64}Au_{36}$/C	1.0 M CH_3OH	1.0 M KOH	-	50	0.50 V	442.3	[11]
Pt@MoS_2-NrGO	1.0 M CH_3OH	0.5 M H_2SO_4	72.33 m^2g^{-1}	50	0.45 V	448 m^2 g^{-1}	[12]
$Pt_{2.6}Co$/C	1.0 M CH_3OH	0.5 M H_2SO_4	24.38 m^2 g^{-1}	50	0.58 V	344 m^2 g^{-1}	[13]
Ru–Pt/Naf Zeo/GC	0.5 M CH_3OH	0.5 M H_2SO_4	-	50	0.58 V	148	[14]
Pt_2/Co_8-NC	0.5 M CH_3OH	0.5 M H_2SO_4	20 m^2 $g_{Pt}{}^{-1}$	50	0.56 V	445 m^2 $g_{Pt}{}^{-1}$	[15]
$Pt_3Ru/Ti_{0.7}W_{0.3}O_2$	1.0 M CH_3OH	0.5 M H_2SO_4	82.12 (m^2 $g_{Pt}{}^{-1}$)	25	0.10 V	350.84 (m^2 $g_{Pt}{}^{-1}$)	[16]
**PtNDs@CoS_2-NrGO**	**1.0 M CH_3OH**	**0.5 M H_2SO_4**	**42.86 m^2g^{-1}**	**50**	**0.35 V**	**491.31 mA $mg_{Pt}{}^{-1}$**	**This work**

Table S7. Comparison table of ethylene glycol (EG) oxidation reaction (EGOR) performances on PtNDs@CoS_2-NrGO and recently reported nanocomposites in both alkaline and acid media.

**Electrocatalyst**	**Fuel**	**Electrolyte**	**ECSA**	**Scan rate (mV s^{-1})**	**Onset potential**	**Peak current density**	**references**
$Pd_{30}Ag_{70}$(100) face sets	0.5 M EG	1.0 M KOH	-	20	-0.41 V	84 mA cm^{-2}	[17]
Pd/C-CeO_2 – 1:1	1.0 M EG	1.0 M KOH	32 $m^2g^{-1}{}_{pd}$	50	-0.37 V	68.5 mA cm^{-2}	[18]
PtRhNi	1.0 M EG	0.3 M KOH	14.47 m^2g^{-1}	50	-0.40 V	79.01 mA cm^{-2}	[19]
Pd/N-Se-C	0.5 M EG	0.1 M KOH	212.13 cm^2mg^{-1}	50	-0.46 V	440 mA $mg^{-1}{}_{Pd}$	[20]
Pd/C	0.5 M EG	0.1 M KOH	83.38 cm^2mg^{-1}	50	-0.26 V	300 mA $mg^{-1}{}_{Pd}$	[20]
Pt/TiN@NrGO (R=14)	0.5 M EG	0.5 M H_2SO_4	54.9 $m^2g^{-1}{}_{Pt}$	50	-0.35 V	47.9 mA $mg^{-1}{}_{Pt}$	[21]
Pt_3Mn-Ru	0.5 M EG	0.1 M $HCLO_4$	18.3 $m^2g^{-1}{}_{Pt}$	50	0.80 V	241.6 mA $mg^{-1}{}_{Pt}$	[22]
PtPd@Pt NCS/rGO	0.5 M EG	0.5 M H_2SO_4	62.19 $m^2g^{-1}{}_{Pt}$	50	0.38 V	230 mA $mg^{-1}{}_{Pt}$	[23]
$Pt_{69}Co_{31}$ NSNS	0.5 M EG	0.5 M H_2SO_4	20.39 m^2g^{-1}	50	0.47 V	85.23 mA cm^{-2}	[24]
Pt_2Mo_1/C	1.0 M EG	0.5 M H_2SO_4	20.39 m^2g^{-1}	50	0.69 V	112 mA cm^{-2}	[25]
**PtNDs@CoS_2-NrGO**	**1.0 M EG**	**0.5 M H_2SO_4**	**42.86 m^2g^{-1}**	**50**	**0.36 V**	**440.25 mA $mg_{Pt}{}^{-1}$**	**This work**

Table S8. Comparison table of glycerol (Gly) oxidation reaction (GOR) performances of PtNDs@CoS_2-NrGO electrocatalyst and recently reported nanocomposites in both alkaline and acid media.

Electrocatalyst	Fuel	Electrolyte	ECSA	Scan rate (mV s^{-1})	Onset potential	Peak current density	references
Pt-Ni Co_2O_4/HPG	1.0 M Gly	1 M KOH	53.8 m^2g^{-1}	5	-0.648 V	48.2 mA cm^{-2}	[26]
Pt Ag NCs	0.5 M Gly	0.5 M KOH	53.87 m^2g^{-1}	50	-	77.91 mA cm^{-2}	[27]
Pd_4Bi	0.1 M Gly	0.1 M KOH	-	100	0.6 V	125.05 mA cm^{-2}	[28]
$Pd_{50}Ag_{50}$/C	0.1 M Gly	0.1 M NaOH	72 $m^2g_{pd}^{-1}$	50	0.4 V	260 A g^{-1}_{Pd}	[29]
$Ni_{90}Bi_{10}$	0.1 M Gly	0.1 M KOH	-	50	-0.45 V	92.6 mA cm^{-2}	[30]
Pt_5Ru_5/C	2.0 M Gly	0.5 M H_2SO_4	-	50	0.448 V	38.6 mA cm^{-2}	[31]
Pt@MoS_2/NrGO	1.0 M Gly	0.5 M H_2SO_4	72.33 $m^2g^{-1}{}_{Pt}$	50	-	147 mA $mg^{-1}{}_{Pt}$	[12]
$Pt_5Ru_4Sn_1$/C	2.0 M Gly	0.5 M H_2SO_4	-	50	0.476 V	87.5 mA cm^{-2}	[32]
$Pt_{69}Co_{31}$ NSNS	0.5 M Gly	0.5 M H_2SO_4	-	20	0.43 V	5.08 mA cm^{-2}	[33]
**PtNDs@CoS_2-NrGO**	**1.0 M Gly**	**0.5 M H_2SO_4**	**42.86 m^2g^{-1}**	**50**	**0. 39 V**	**438.12 mA mg_{Pt}^{-1}**	**This work**

Table S9. Calculated surface and adsorption energies of Pt (111) and CoS_2 (200) surfaces from DFT calculation.

Composite	Surface energy, γ (J/m^2)	Adsorption energy, E_{ads} for CH_3OH molecule (eV)
Pt (111)	1.45	-0.49
CoS_2 (200)	0.47	-0.14

Table S10. ICOHP values of methanol adsorbed CoS_2 (200) surface with different atomic interactions in up and down spin channels.

Interactions in methanol adsorbed CoS_2 (200) surface	-ICOHP
Co-C (Up spin)	0.0029
Co-C (Down spin)	0.0006
Co-O (Up spin)	0.0215
Co-O (Down spin)	0.0267
Co-H (Up spin)	0.0043
Co-H (Down spin)	0.0015
S-C (Up spin)	0.0032
S-C (Down spin)	0.0029
S-O (Up spin)	0.0068
S-O (Down spin)	0.0062
S-H (Up spin)	0.0269
S-H (Down spin)	0.0268

References

[1] G. Kresse, J. Furthmüller, *Phys. Rev. B* **1996**, *54* (16), 11169, https://doi.org/10.1103/PhysRevB.54.11169.

[2] G. Kresse, D. Joubert, *Phys. Rev. B* **1999**, *59* (3), 1758, https://doi.org/10.1103/PhysRevB.59.1758.

[3] P. E. Blöchl, *Phys. Rev. B* **1994**, *50* (24), 17953, https://doi.org/10.1103/PhysRevB.50.17953.

[4] J. P. Perdew, K. Burke, M. Ernzerhof, *Phys. Rev. Lett.* **1996**, *77* (18), 3865, https://doi.org/10.1103/PhysRevLett.77.3865.

[5] *Q. Rev. Biophys.* **1988**, *21* (2), b1, https://doi.org/10.1017/S0033583500004285.

[6] E. P. Lee, Z. Peng, D. M. Cate, H. Yang, C. T. Campbell, Y. Xia, *J. Am. Chem. Soc.* **2007**, *129* (35), 10634, https://doi.org/10.1021/ja074312e.

[7] T. Jurzinsky, R. Bär, C. Cremers, J. Tübke, P. Elsner, *Electrochim. Acta* **2015**, *176*, 1191, https://doi.org/https://doi.org/10.1016/j.electacta.2015.07.176.

[8] J. Li, Z. Luo, Y. Zuo, J. Liu, T. Zhang, P. Tang, J. Arbiol, J. Llorca, A. Cabot, *Appl. Catal., B* **2018**, *234*, 10, https://doi.org/https://doi.org/10.1016/j.apcatb.2018.04.017.

[9] Y.-Y. Feng, G.-H. Song, Q. Zhang, H.-S. Hu, M.-Y. Feng, J.-Y. Wang, D.-S. Kong, *Int. J. Hydrogen Energy* **2017**, *42* (51), 30109, https://doi.org/https://doi.org/10.1016/j.ijhydene.2017.10.102.

[10] Q.-Y. Hu, L.-M. Luo, R.-H. Zhang, D. Chen, Y.-F. Guo, W. Zhan, X.-W. Zhou, *J. Alloys Compd.* **2020**, *818*, 152886, https://doi.org/https://doi.org/10.1016/j.jallcom.2019.152886.

[11] Z. Yin, M. Chi, Q. Zhu, D. Ma, J. Sun, X. Bao, *J. Mater. Chem. A* **2013**, *1* (32), 9157, https://doi.org/10.1039/C3TA11592E.

[12] S. Ramakrishnan, M. Karuppannan, M. Vinothkannan, K. Ramachandran, O. J. Kwon, D. J. Yoo, *ACS Appl. Mater. Interfaces* **2019**, *11* (13), 12504, https://doi.org/10.1021/acsami.9b00192.

[13] X. Wang, C. Yang, L. Cao, H.-P. Liang, *New J. Chem.* **2020**, *44* (15), 5792, https://doi.org/10.1039/D0NJ00242A.

[14] M. I. Awad, W. S. Al-Saidi, M. S. El-Deab, B. A. Al-Jahdaly, M. A. Kassem, *Int. J. Hydrogen Energy* **2020**, *45* (51), 27171, https://doi.org/https://doi.org/10.1016/j.ijhydene.2020.07.056.

[15] W. Ren, W. Zang, H. Zhang, J. Bian, Z. Chen, C. Guan, C. Cheng, *Carbon* **2019**, *142*, 206, https://doi.org/https://doi.org/10.1016/j.carbon.2018.10.054.

[16] H. Q. Pham, T. T. Huynh, T. M. Pham, V. T. T. Ho, *Int. J. Hydrogen Energy* **2020**, https://doi.org/https://doi.org/10.1016/j.ijhydene.2020.08.278.

[17] A. López–Coronel, E. Ortiz–Ortega, L. J. Torres–Pacheco, M. Guerra–Balcázar, L. G. Arriaga, L. Álvarez–Contreras, N. Arjona, *Electrochim. Acta* **2019**, *320*, 134622, https://doi.org/https://doi.org/10.1016/j.electacta.2019.134622.

[18] S. Sankar, N. Watanabe, G. M. Anilkumar, B. N. Nair, S. G. Sivakamiammal, T. Tamaki, T. Yamaguchi, *Catal. Sci. Technol.* **2019**, *9* (2), 493, https://doi.org/10.1039/C8CY02232A.

[19] V. L. Marinho, L. A. Pocrifka, R. R. Passos, *J. Solid State Electrochem.* **2018**, *22* (5), 1517, https://doi.org/10.1007/s10008-017-3825-7.

[20] Y. Dong, J. Zhao, J. Han, Y. Kang, Y. Mi, W. Wang, *J. Mater. Sci. Eng. B* **2020**, *252*, 114467, https://doi.org/https://doi.org/10.1016/j.mseb.2019.114467.

[21] T. Yang, C. Zhang, Z. Wang, P. Bai, X. Wang, Z. Chai, *Adv. Mater. Interfaces* **2020**, *7* (20), 2000808, https://doi.org/https://doi.org/10.1002/admi.202000808.

[22] Y. Wang, M. Zheng, H. Sun, X. Zhang, C. Luan, Y. Li, L. Zhao, H. Zhao, X. Dai, J.-Y. Ye, H. Wang, S.-G. Sun, *Appl. Catal., B* **2019**, *253*, 11, https://doi.org/https://doi.org/10.1016/j.apcatb.2019.04.022.

[23] L. Liu, X.-X. Lin, S.-Y. Zou, A.-J. Wang, J.-R. Chen, J.-J. Feng, *Electrochim. Acta* **2016**, *187*, 576, https://doi.org/https://doi.org/10.1016/j.electacta.2015.11.089.

[24] Z.-N. Yu, Z. Zhang, Z.-S. Lv, M.-T. Liu, L. Zhang, A.-J. Wang, L.-Y. Jiang, J.-J. Feng, *J. Colloid Interface Sci.* **2018**, *525*, 216, https://doi.org/https://doi.org/10.1016/j.jcis.2018.04.050.

[25] W. J. Pech-Rodríguez, C. Calles-Arriaga, D. González-Quijano, G. Vargas-Gutiérrez, C. Morais, T. W. Napporn, F. J. Rodríguez-Varela, *J. Power Sources* **2018**, *375*, 335, https://doi.org/https://doi.org/10.1016/j.jpowsour.2017.07.081.

[26] B.-C. Liu, S.-L. Chen, X.-Y. Ling, Q.-X. Li, C.-W. Xu, Z.-L. Liu, *RSC Adv.* **2020**, *10* (41), 24705, https://doi.org/10.1039/C9RA09896H.

[27] X. Weng, Q. Liu, A.-J. Wang, J. Yuan, J.-J. Feng, *J. Colloid Interface Sci.* **2017**, *494*, 15, https://doi.org/https://doi.org/10.1016/j.jcis.2017.01.030.

[28] A. Zalineeva, A. Serov, M. Padilla, U. Martinez, K. Artyushkova, S. Baranton, C. Coutanceau, P. B. Atanassov, *J. Am. Chem. Soc.* **2014**, *136* (10), 3937, https://doi.org/10.1021/ja412429f.

[29] Y. Holade, C. Morais, K. Servat, T. W. Napporn, K. B. Kokoh, *ACS Catal.* **2013**, *3* (10), 2403, https://doi.org/10.1021/cs400559d.

[30] M. S. E. Houache, K. Hughes, R. Safari, G. A. Botton, E. A. Baranova, *ACS Appl. Mater. Interfaces* **2020**, *12* (13), 15095, https://doi.org/10.1021/acsami.9b22378.

[31] Y. Kim, H. W. Kim, S. Lee, J. Han, D. Lee, J.-R. Kim, T.-W. Kim, C.-U. Kim, S.-Y. Jeong, H.-J. Chae, B.-S. Kim, H. Chang, W. B. Kim, S. M. Choi, H. J. Kim, *ChemCatChem* **2017**, *9* (9), 1683, https://doi.org/https://doi.org/10.1002/cctc.201601325.

[32] H. J. Kim, S. M. Choi, S. Green, G. A. Tompsett, S. H. Lee, G. W. Huber, W. B. Kim, *Appl. Catal., B* **2011**, *101* (3), 366, https://doi.org/https://doi.org/10.1016/j.apcatb.2010.10.005.

[33] L. S. R. Silva, F. E. López-Suárez, M. Perez-Cadenas, S. F. Santos, L. P. da Costa, K. I. B. Eguiluz, G. R. Salazar-Banda, *Appl. Catal., B* **2016**, *198*, 38, https://doi.org/https://doi.org/10.1016/j.apcatb.2016.04.046.
